# Coordination of Hepatitis C Virus Assembly by Distinct Regulatory Regions in Nonstructural Protein 5A

**DOI:** 10.1371/journal.ppat.1005376

**Published:** 2016-01-04

**Authors:** Margarita Zayas, Gang Long, Vanesa Madan, Ralf Bartenschlager

**Affiliations:** Department for Infectious Diseases, Molecular Virology, Heidelberg University, Heidelberg, Germany; The Scripps Research Institute, UNITED STATES

## Abstract

Hepatitis C virus (HCV) nonstructural protein (NS)5A is a RNA-binding protein composed of a N-terminal membrane anchor, a structured domain I (DI) and two intrinsically disordered domains (DII and DIII) interacting with viral and cellular proteins. While DI and DII are essential for RNA replication, DIII is required for assembly. How these processes are orchestrated by NS5A is poorly understood. In this study, we identified a highly conserved basic cluster (BC) at the N-terminus of DIII that is critical for particle assembly. We generated BC mutants and compared them with mutants that are blocked at different stages of the assembly process: a NS5A serine cluster (SC) mutant blocked in NS5A-core interaction and a mutant lacking the envelope glycoproteins (ΔE1E2). We found that BC mutations did not affect core-NS5A interaction, but strongly impaired core–RNA association as well as virus particle envelopment. Moreover, BC mutations impaired RNA-NS5A interaction arguing that the BC might be required for loading of core protein with viral RNA. Interestingly, RNA-core interaction was also reduced with the ΔE1E2 mutant, suggesting that nucleocapsid formation and envelopment are coupled. These findings argue for two NS5A DIII determinants regulating assembly at distinct, but closely linked steps: (i) SC-dependent recruitment of replication complexes to core protein and (ii) BC-dependent RNA genome delivery to core protein, triggering encapsidation that is tightly coupled to particle envelopment. These results provide a striking example how a single viral protein exerts multiple functions to coordinate the steps from RNA replication to the assembly of infectious virus particles.

## Author Summary

Hepatitis C virus (HCV) nonstructural protein (NS)5A is an enigmatic RNA-binding protein that appears to regulate the different steps from RNA replication to the assembly of infectious virus particles by yet unknown mechanisms. Assembly requires delivery of the viral RNA genome from the replication machinery to the capsid protein to ensure genome packaging into nucleocapsids that acquire a membranous envelope by budding into the lumen of the endoplasmic reticulum. In this study, we provide genetic and biochemical evidence that the viral nonstructural protein (NS)5A contains two regulatory determinants in its domain (D)III that orchestrate virus assembly at two closely linked steps: (i) recruitment of replication complexes to core protein requiring a serine cluster in the C-terminal region of DIII and (ii) RNA genome delivery to core protein requiring a basic cluster in the N-terminal region of DIII. This RNA transfer most likely triggers encapsidation, which is tightly coupled to particle envelopment. These results provide a striking example for a multi-purpose viral protein exerting several distinct functions in the viral replication cycle, thus reflecting genetic economy.

## Introduction

The hepatitis C virus (HCV) is a major causative agent of chronic liver diseases, affecting ~170 million people worldwide. HCV infection is frequently asymptomatic, however persistently infected people have a high risk to develop serious liver diseases including liver cirrhosis and hepatocellular carcinoma [1]. HCV belongs to the genus *Hepacivirus* within the family *Flaviviridae*, which is a group of enveloped viruses with a single strand RNA genome of positive polarity. The HCV genome has a length of ~9,600 nucleotides and contains one long open reading frame that is flanked at the 5’ and 3’ end by non-translated regions required for RNA translation and replication, respectively [2]. The open reading frame encodes a polyprotein precursor of ~3,000 amino acids that is translated at the rough endoplasmic reticulum (ER). Polyprotein cleavage occurs co- and post-translationally and is mediated by cellular and viral proteases giving rise to at least 10 different products: three structural proteins that build up the virus particle [core, envelope protein 1 (E1) and E2], and seven nonstructural proteins, i.e. p7, NS2, NS3, NS4A, NS4B, NS5A and NS5B. The viroporin p7 and the cysteine protease NS2 are not essential for RNA replication, but are required for the assembly of infectious HCV particles [3–6]. The remaining proteins NS3 to NS5B form a membrane-associated replicase complex catalyzing viral RNA replication [2].

Like all positive-strand RNA viruses, HCV induces intracellular membrane rearrangements giving rise to cytoplasmic replication factories that are composed primarily of ER-derived double membrane vesicles (DMVs) [7], the presumed sites of RNA replication [8]. Newly synthesized viral RNA can be used for RNA translation, and thus production of viral proteins, or it is packaged into virus particles. Their assembly occurs in close proximity of cytosolic lipid droplets (cLDs) [9] and includes genome encapsidation as well as acquisition of a membranous envelope by budding into the ER lumen [10]. This process and the secretion of HCV particles require components of the machinery responsible for very-low-density lipoprotein (VLDL) formation (reviewed in [11]). In fact, released virions contain apolipoproteins and HCV particles contained in patient serum [12] or produced in cell culture [13–17] have an unusually low buoyant density ranging between 1.006–1.055 g/ml and 1.1–1.15 g/ml, respectively. This biophysical property is consistent with the lipid composition of HCV particles that contain high amounts of cholesterol and cholesterol esters [18]. This might be due to the formation of hybrid lipo-viral particle (LPV) [12] or to tight interaction of virions with VLDL/LDL-particles [19].

Assembly of infectious virus particles requires the coordinated packaging of the viral RNA genome into the nucleocapsid. It is composed of multiple copies of the core protein possessing three domains: domain (D)1 interacting with viral RNA and forming homo-oligomers [20–22]; D2 targeting the protein to cLDs [23–26]; D3 serving as signal sequence that targets the nascent polyprotein to the ER membrane [27]. Trafficking of core to cLDs requires the removal of D3 by signal peptide peptidase, which is an essential step for virus particle production [28, 29]. Apart from core protein, NS5A is the only other protein that also traffics to cLDs where it colocalizes with core and this colocalization appears to be required for the assembly of infectious HCV particles [9, 30, 31]. NS5A is a multifunctional RNA-binding phosphoprotein forming homodimers and eventually also oligomeric complexes [32, 33]. NS5A is composed of a N-terminal amphipathic α-helix (AH) that anchors the protein to intracellular membranes [34] and three domains that are separated by two low complexity sequences (LCS) [35]. DI is a highly structured domain essential for RNA replication and engaged in self-interaction [33] whereas DII and DIII are natively unfolded domains [36, 37]. All domains of NS5A were reported to interact with HCV RNA: predominantly DI, and to a lesser extent DII and DIII [38, 39]. We and others have found that NS5A DIII, in particular a serine cluster (SC) in its C-terminal region is required for particle assembly [30, 31, 40]. Alanine substitutions in the SC disrupt core-NS5A interaction and cause an accumulation of NS5A around cLDs that are devoid of core protein [30, 31]. Based on these results it has been proposed that NS5A might regulate the switch between replication of the viral RNA and its packaging into virions [11, 19]. However, the mechanism by which NS5A exerts this regulation is poorly understood.

In the present study we conducted a comparative analysis of NS5A mutations causing an arrest of the assembly process at different stages. We report a novel basic cluster (BC) at the N-terminus of NS5A DIII interacting with viral RNA and required for loading of the core protein with the RNA genome. This process likely triggers formation of nucleocapsids, which is linked to the envelopment of virus particles.

## Results

### A basic cluster motif in domain III of NS5A is required for efficient production of infectious HCV particles

**Fig 1:**
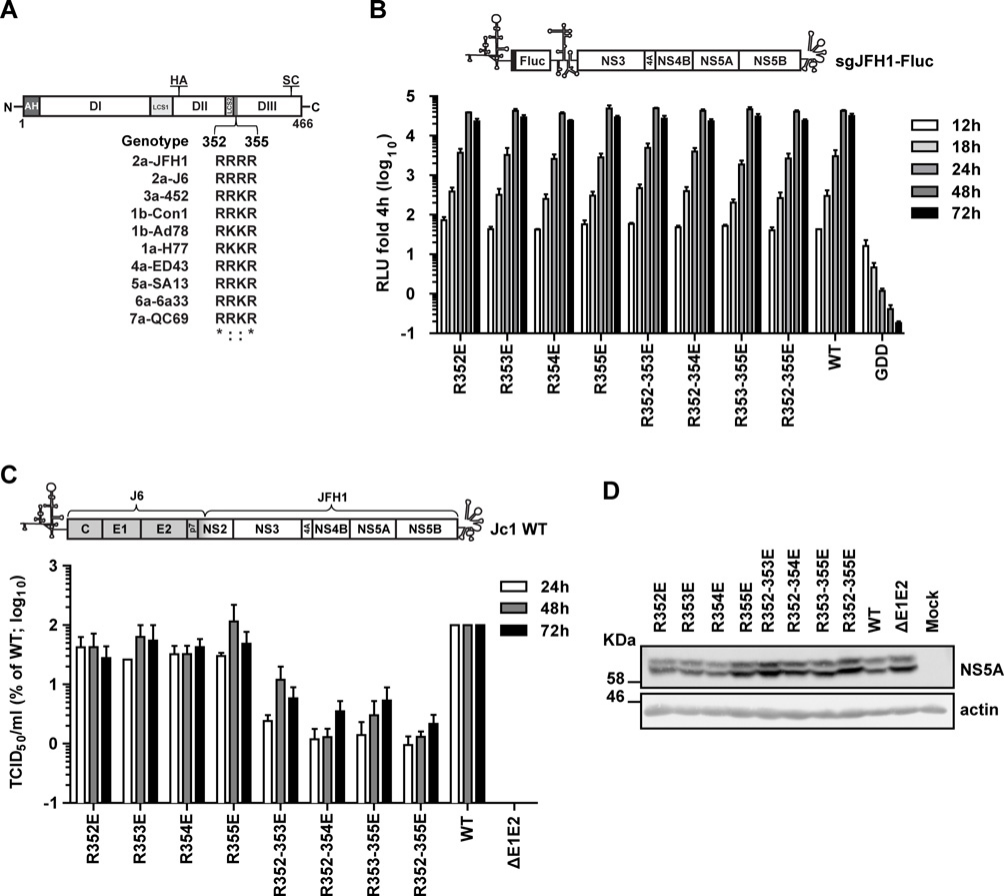
Impact of mutations affecting the basic cluster motif in NS5A DIII on RNA replication and virus production. (A) Schematic representation of NS5A domains: amphipathic α-helix (AH); domain (D) I, II and III; low complexity sequences (LCS) 1 and 2. The positions of the hemagglutinin (HA) epitope tag inserted within DII and the serine cluster (SC; S452/454/457) in DIII are indicated on the top. An alignment of the amino acid sequence of the NS5A basic cluster motif of several HCV isolates belonging to genotype 1 to 7 is given at the bottom. Numbers refer to amino acid residues 352 to 355 of the JFH1 isolate (corresponding to polyprotein residues 2328 to 2331). *, invariant amino acid residue across the displayed HCV isolates;:, conservation of physicochemical properties of the amino acid. The following HCV genomes were used for the alignment (gene bank accession numbers are given in parenthesis): H77 (AF009606), Con1 (AJ238799), Ad78 (AJ132997), J6 2a (Af177036), 452 (DQ437509), ED43 (Y11604), SA13 (AF064490), 6a33 (AY859526), QC69 (EF108306) and JFH1. (B) Given glutamic acid residue substitutions were inserted into a subgenomic JFH1 Firefly luciferase reporter replicon (sgJFH1-Fluc; top panel) and replication kinetics were determined. The various sgJFH1 constructs were transfected into Huh7-Lunet cells and harvested 4, 12, 18, 24, 48 and 72 h later. Luciferase activity was quantified and values were normalized to the respective 4 h-value. Mean and SEM of three independent experiments are shown. Background was determined with an RdRp-defective mutant (GDD). (C) The same mutations were introduced into the full-length HCV chimera Jc1 [4] shown in the top and 24, 48 and 72 h after transfection into Huh7-Lunet cells virus amounts contained in culture supernatants were quantified by limiting dilution assay. Values were normalized to the wildtype (WT) virus that was set to 100%. Mean and SEM of three independent experiments are shown. Background of the assay was determined with a deletion mutant lacking the envelope glycoprotein coding region (ΔE1E2). (D) NS5A amounts contained in cells 72 h after transfection with the Jc1 variants were determined by Western Blot with NS5A-specific antibodies; ß-actin served as loading control. Numbers in the left refer to apparent molecular weights of marker proteins in kilo Dalton (KDa).

We have previously reported that deletions in NS5A DIII impaired particle production [30]. However we noticed that particle production was completely abrogated only when the N-terminal region of DIII was included in the deletions that we had analyzed (amino acid residues 352–459 corresponding to residues 2328–2435 of the JFH-1 polyprotein) [30]. This result argued for a critical assembly determinant in this NS5A region and we addressed our assumption by searching for highly conserved amino acid sequence motifs in this site. A comparison of NS5A sequences between well-characterized HCV strains belonging to genotype 1–7 revealed a highly conserved stretch of four basic amino acid residues in the N-terminal region of DIII (Fig 1A). To determine the role of this “basic cluster” (BC) motif for the viral replication cycle, we generated several glutamic acid residue substitutions that were introduced into the JFH1-derived subgenomic luciferase reporter replicon (sgJFH1-Fluc) (Fig 1B). *In vitro* transcripts of this replicon were transfected into Huh7-Lunet cells and RNA replication was monitored by determining luciferase activity 4, 12, 18, 24, 48 and 72 h post transfection. As shown in Fig 1B, none of the mutations affected RNA replication kinetics. To assess the impact of the NS5A BC mutations on virus particle production, they were inserted into the highly assembly competent HCV variant Jc1 [4]. *In vitro* transcripts were transfected into Huh7-Lunet cells and amounts of infectious virus particles released into the culture supernatants 24, 48 and 72 h after transfection were determined by limiting dilution assay. The results presented in Fig 1C show that infectivity titers of the single point mutants did not differ significantly from the wildtype whereas no infectivity was released from cells transfected with an envelope glycoprotein deletion mutant (ΔE1E2) that served as negative control. However, by gradually changing the BC into an acidic one, particle production was profoundly impaired, which was most pronounced in case of the quadruple mutant R352-355E that released ~100-fold lower amounts of infectious virus particles as compared to the wildtype. This reduction was not due to altered stability of NS5A proteins, because comparable amounts were detected in the Jc1-transfected cells (Fig 1D). Importantly, the reduction of virus production was not caused by the negative charge introduced into the BC, because alanine substitutions at the same site also reduced virus production, although the degree of impairment was lower as compared to the quadruple glutamic acid substitution (10-fold versus 100-fold, respectively; S1 Fig). Moreover, this phenotype was not genotype specific, since glutamic acid substitutions in the conserved BC motif of the infectious genotype 1a strain H77S [16, 41] also reduced production of infectious HCV (S2 Fig). Taken together, these results suggested that the BC in NS5A DIII is involved in the production of infectious HCV particles in a genotype-independent manner.

**Fig 2:**
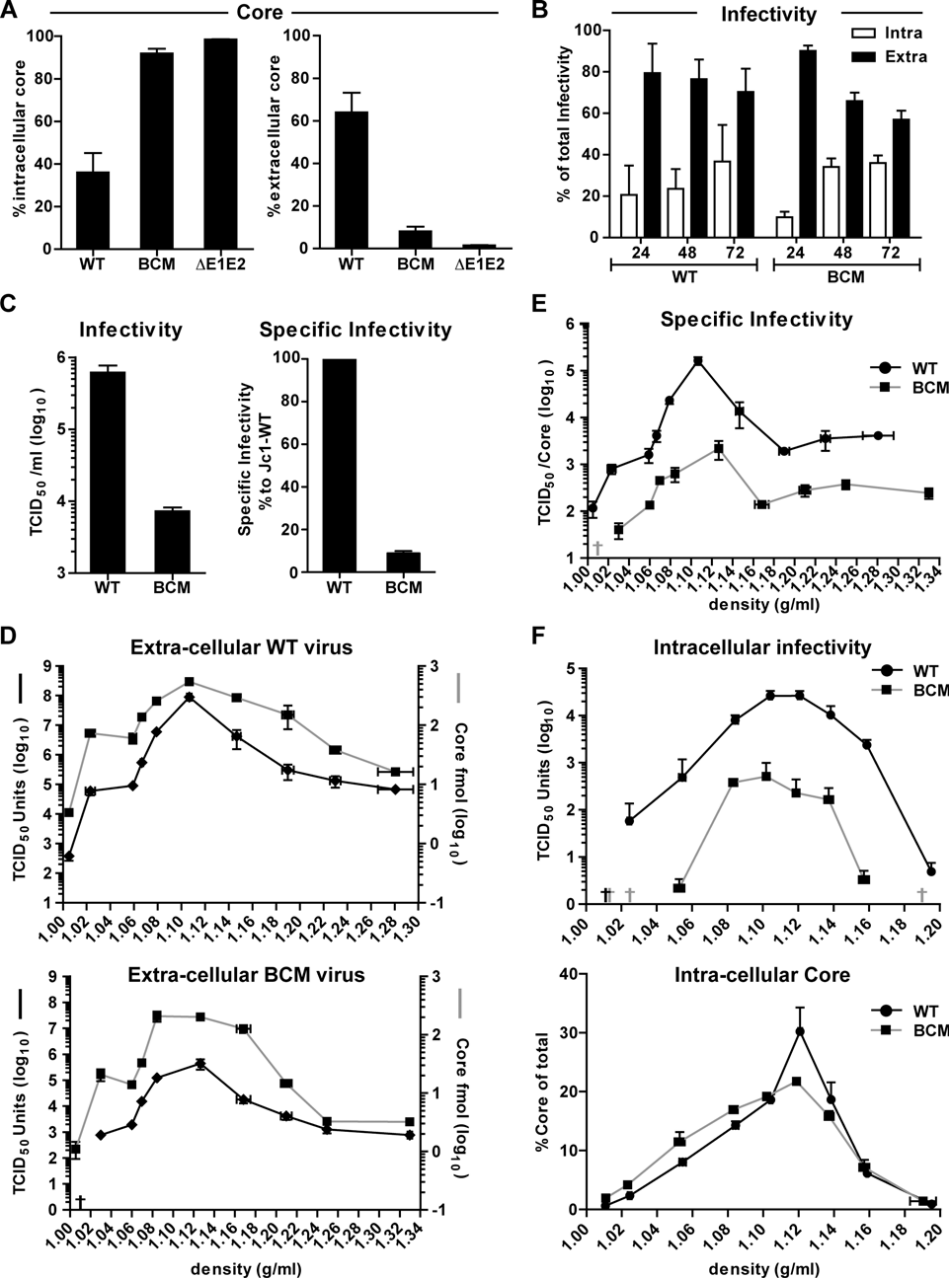
Mutations disrupting the basic cluster in NS5A DIII impair assembly and release of infectious HCV particles. Huh7-Lunet cells were transfected with genomic RNAs of Jc1 wildtype (WT), NS5A basic cluster mutant R352-355E (BCM) and a Jc1 genome lacking the envelope glycoproteins E1 and E2 coding region (ΔE1E2). Four and 48 h post transfection, cells and supernatants were harvested and titers of infectious virus as well as core amounts were determined by limiting dilution assay and CMIA, respectively. (A) For each HCV construct, intra- and extra-cellular amounts of core (normalized to total core amount that was set to 100%) are given (left and right panel, respectively). Mean and SEM of three independent experiments are shown. (B) Huh7-Lunet cells were transfected with HCV RNA genomes specified in the bottom and 24, 48 and 72 h later, amounts of infectious virus contained in cell lysates and culture supernatant were determined by limiting dilution assay. For each time point the relative amounts of intra- and extracellular infectivity are given. Mean and SEM of three independent experiments are shown. (C) Titers of infectious virus and corresponding specific infectivities (left and right panel, respectively). Specific infectivity was determined by calculating the ratio of infectivity (TCID_50_/ml) and core protein amount (pg/ml) and normalization to the wildtype that was set to 100%. Mean and SEM of two independent experiments are shown. (D) Density distribution of infectious virus particles released into culture supernatants of cells transfected with the Jc1 wildtype or the NS5A basic cluster mutant (upper and lower panel, respectively). Supernatants were collected 48 and 72 h post transfection, pooled, concentrated, loaded on top of a 20–80% Optiprep density gradient and subjected to equilibrium ultracentrifugation. Ten fractions were collected and amounts of infectivity contained in each fraction were determined. (E) Specific infectivity contained in each fraction shown in panel (D) was calculated as described above. Mean and SEM of two independent experiments is shown. (F) Density distribution of virus particles contained in cells 48 h after transfection. Pre-cleared lysates were loaded on top of a 20–60% Optiprep density gradient and subjected to equilibrium ultracentrifugation. Infectivity titers and core protein amounts contained in each fraction are shown in the upper and lower panels, respectively. Mean and SEM of two independent experiments is shown. Values below the detection limit of the TCID_50_ assay are indicated with crosses.

Next we determined whether reduced virus amounts were due to defects in assembly, impaired release of virus particles or reduced particle infectivity. To this end, core protein amounts contained in cells and released into the culture supernatants 48 h after transfection with the Jc1 wildtype, the ΔE1E2 deletion mutant or the NS5A BC mutant R352-355E (from now on referred as BC mutant) were determined by using a core-specific chemiluminescence-based immunoassay (CMIA). Cells transfected with the wildtype released ~60% of total core protein (i.e. intra- plus extracellular core) (Fig 2A). In contrast, the NS5A BC mutant and the envelope glycoprotein deletion mutant ΔE1E2 released only ~10% and ~2% of total core protein, respectively (Fig 2A, left and right panel, respectively). Thus, at least part of the defect of the NS5A BC mutant was due to impaired assembly/release of HCV particles.

To rule out that the block in core release was due to impairment in secretion of viral particles, we determined the total production of infectious virions (i.e. the sum of intra- and extracellular infectivity), at different time points after transfection and calculated the ratio between intra- and extracellular infectivity. No major differences were observed at any time point post transfection between wildtype and the NS5A BC mutant (Fig 2B), arguing that the defect observed was primarily at the level of particle assembly (Fig 2B). Furthermore, when we calculated the specific infectivity (infectious units per core protein quantity) of the virus particles produced by the NS5A BC mutant, we determined a reduction of ~90% as compared to the wildtype (Fig 2C). This observation was corroborated when we analyzed the NS5A BC mutation in the context of a Jc1 genome containing a Flag tag at the N-terminus of E2 to allow affinity purification of HCV particles (S3A Fig) [18]. Consistent with the bulk measurement, specific infectivity of the E2^Flag^-captured particles was significantly reduced in case of the NS5A BC mutant as deduced from a comparison of the ratio between TCID_50_ units per picogram of core protein or per HCV RNA copies (S3B and S3C Fig, respectively). Interestingly, we noticed that in contrast to the WT infectivity of the virus particles produced by the NS5A BCM was reduced after affinity purification (S3A Fig, right panel), arguing that these viral particles might be less stable; however, this assumption remains to be validated. In summary, these results suggest that the BC is not only involved in the assembly process, but also determines the infectivity of progeny virus particles.

To investigate the biophysical properties of BC mutant virus particles, we determined their density profile. Virus particles contained in supernatants and cell lysates 48 h after transfection were subjected to isopycnic density gradient centrifugation and infectivity as well as core protein amounts contained in each fraction were determined. In case of extracellular virus particles, density profiles observed with the NS5A BC mutant and the wildtype were comparable (Fig 2D, upper and lower panel, respectively). However, in case of the BC mutant infectivity titers in most fractions were much stronger reduced relative to core protein amounts as compared to the wildtype. This became best visible when calculating the specific infectivity that was reduced with all gradient fractions (Fig 2E). In case of intracellular HCV particles, as expected overall infectivity was profoundly reduced in case of the BC mutant whereas density profiles covered a broad range and were only slightly different between the wildtype and the mutant (Fig 2F, upper panel). Moreover, in case of the wildtype a pronounced peak of core protein at a density of ~1.12 g/ml was detected, which was lower in case of the BC mutant (Fig 2F, bottom panel). In summary, these results suggested that the biophysical properties of extra- and intracellular virus particles produced by the NS5A BC mutant were not grossly different from the wildtype; the most prominent phenotype was the overall reduction of the specific infectivity of virus particles. The observation that infectivity was reduced also with intracellular particles argued for an assembly defect of the BC mutant.

### Important role of the basic cluster in NS5A DIII for the envelopment of HCV particles

**Fig 3:**
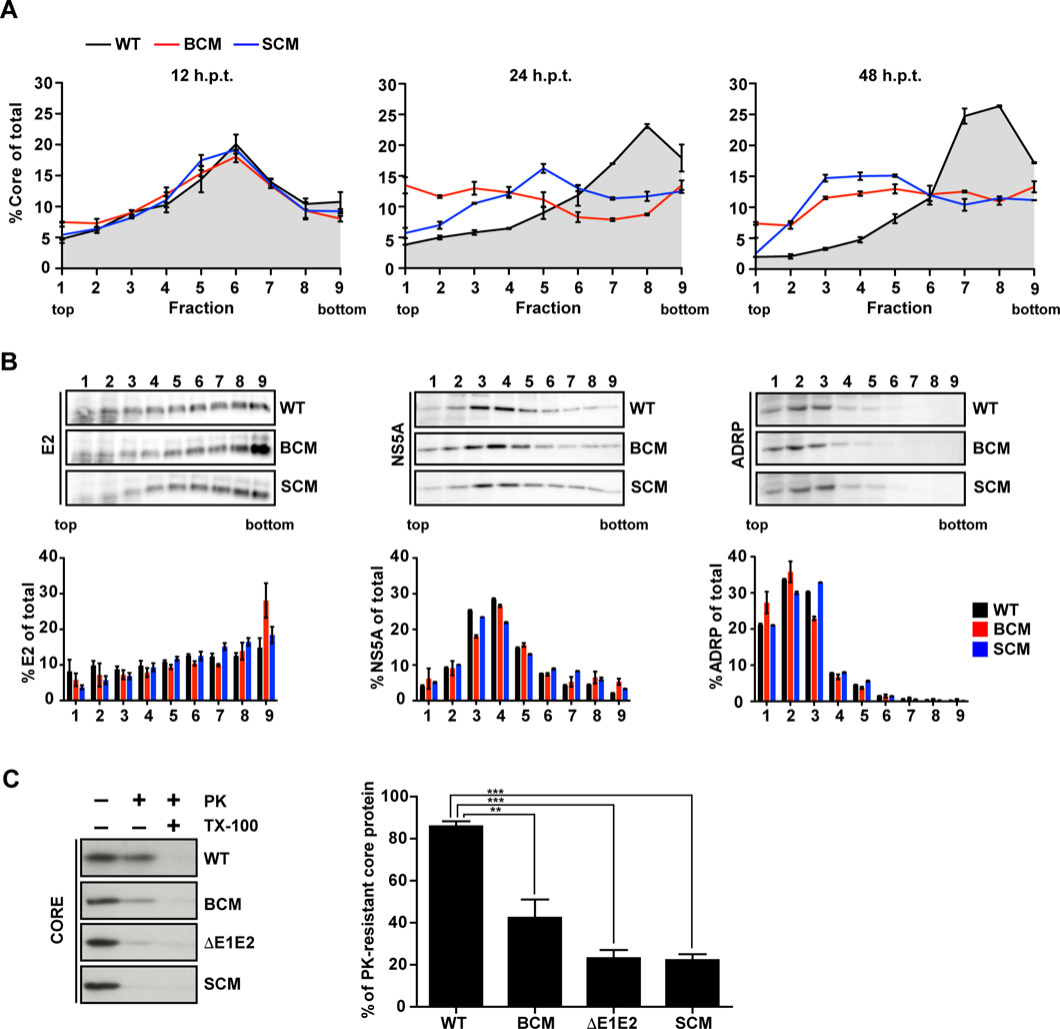
NS5A basic cluster mutant is impaired in core envelopment. (A) Huh7-Lunet cells were transfected with RNA genomes specified in the top: Jc1 wildtype (WT), NS5A R352-355E basic cluster mutant (BCM) and NS5A serine cluster mutant S452/454/457A (SCM). Twelve, 24 and 48 h post transfection cell lysates were prepared and separated by using a 0–30% linear sucrose gradient (left, middle and right panel, respectively). Core protein amounts contained in each fraction were quantified and normalized to total core contained in all fractions. (B) The amounts of E2 (left), NS5A (middle) and ADRP (right) contained in each fraction of cell lysates prepared 48 h post transfection were determined by quantitative Western blot. For each fraction, relative protein amounts are displayed (lower panels). Mean and SEM of two independent experiments are shown. (C) Huh7-Lunet cells were transfected with given HCV genomes and 48 h later cell lysates were prepared and either mock treated or incubated with 15 μg/ml proteinase K (PK) for 40 min on ice. As positive control, samples were treated with 1% Triton X-100 (TX-100) prior to PK digestion. The amount of core protein resistant to PK treatment was determined by Western blot and CMIA (left and right panel, respectively). The graph shows the percentage of PK-resistant core protein. Mean and SEM of three independent experiments is shown. **, *p ≤0*.01; ***, *p ≤0*.*001*.

Assembly of infectious HCV particles requires the acquisition of a lipid membrane containing the envelope glycoproteins. As previously reported, this process can be monitored in a time-resolved manner by determining the sedimentation profile of core protein using rate zonal centrifugation [42]. To this end, we prepared lysates of cells 12, 24 and 48 h after transfection and post-nuclear supernatants were loaded on top of a linear sucrose gradient (0–30%) and separated by rate zonal centrifugation. Gradients were fractionated and core protein amount in each fraction was determined by CMIA (Fig 3A). For comparison we included a NS5A serine cluster mutant (SCM; S452/454/457A; Fig 1A), which owing to a block in NS5A –core interaction has an assembly defect [31]. At 12 h post transfection core complexes of all analyzed HCV constructs sedimented rather slowly with a peak in fractions 5 to 7. In case of the wildtype, already 12 h later a large proportion of core protein had assembled into faster sedimenting complexes and 48 h after transfection up to 70% of total core had assembled into fast-sedimenting complexes that accumulated in fractions 7 to 9. In case of the NS5A BC mutant, the main core peak formed at 12 h also rapidly disappeared, however only 35% of total core protein was found in fast-sedimenting complexes and no distinct peak was detected even 48 h after transfection. Instead, at this time point the amount of core protein in each fraction was rather similar, suggesting that no discrete core protein-containing complex had formed. A similar result was found with the NS5A SC mutant, although at 24 h post transfection the slow-sedimenting core peak did not disappear completely and at 48 h post transfection 33% of total core protein formed slowly sedimenting complexes accumulating in fractions 3 to 5.

To determine the co-sedimentation of core protein with the HCV envelope glycoproteins, gradient fractions of samples harvested 48 h after transfection were analyzed by Western blot. In case of the wildtype ~30% of E2 co-sedimented in the peak fraction of core protein and a similar E2 distribution was found with both NS5A mutants (Fig 3B, left panel). Also the sedimentation profiles of NS5A of wildtype and the mutants were comparable with a peak in fractions 3 to 5 (Fig 3B, middle panel). As expected, the slowly sedimenting adipophilin (ADRP), a bona fide marker protein of lipid droplets (LDs), accumulated in the low density fractions 1 to 5 (Fig 3B, right panel) where also the majority of core protein of the two NS5A mutants had accumulated. These results suggest that core protein-containing complexes formed by the NS5A BC mutant accumulated at LDs as reported earlier for the NS5A SC mutant [31]. This result was consistent with a defect at an early step of HCV assembly leading to an accumulation of (non-assembled) core protein on the surface or in close proximity of LDs [43].

In order to prove that core protein-containing complexes generated by the NS5A BC mutant were not enveloped, we performed a proteinase K (PK) protection assay as previously described [42, 44]. To this end, we collected cells 48 h after transfection and prepared cell lysates by several freeze-and-thaw cycles. Pre-cleared samples were mock-treated or treated with PK in the absence or presence of Triton X-100, which was used to dissolve all lipids to allow complete digest of core protein by PK. Remaining core protein in the different reactions was analyzed by Western blot and quantified by core-specific CMIA. As shown in Fig 3C, in case of the Jc1 wildtype ~80% of core protein was PK resistant, but only ~40% in case of the NS5A BC mutant, arguing for impaired envelopment. As expected, only ~20% of core protein complexes generated by the proven envelopment-deficient ΔE1E2 and the NS5A SC mutant were PK resistant.

### Redistribution of the E2 glycoprotein is not affected by the NS5A BC mutant

**Fig 4:**
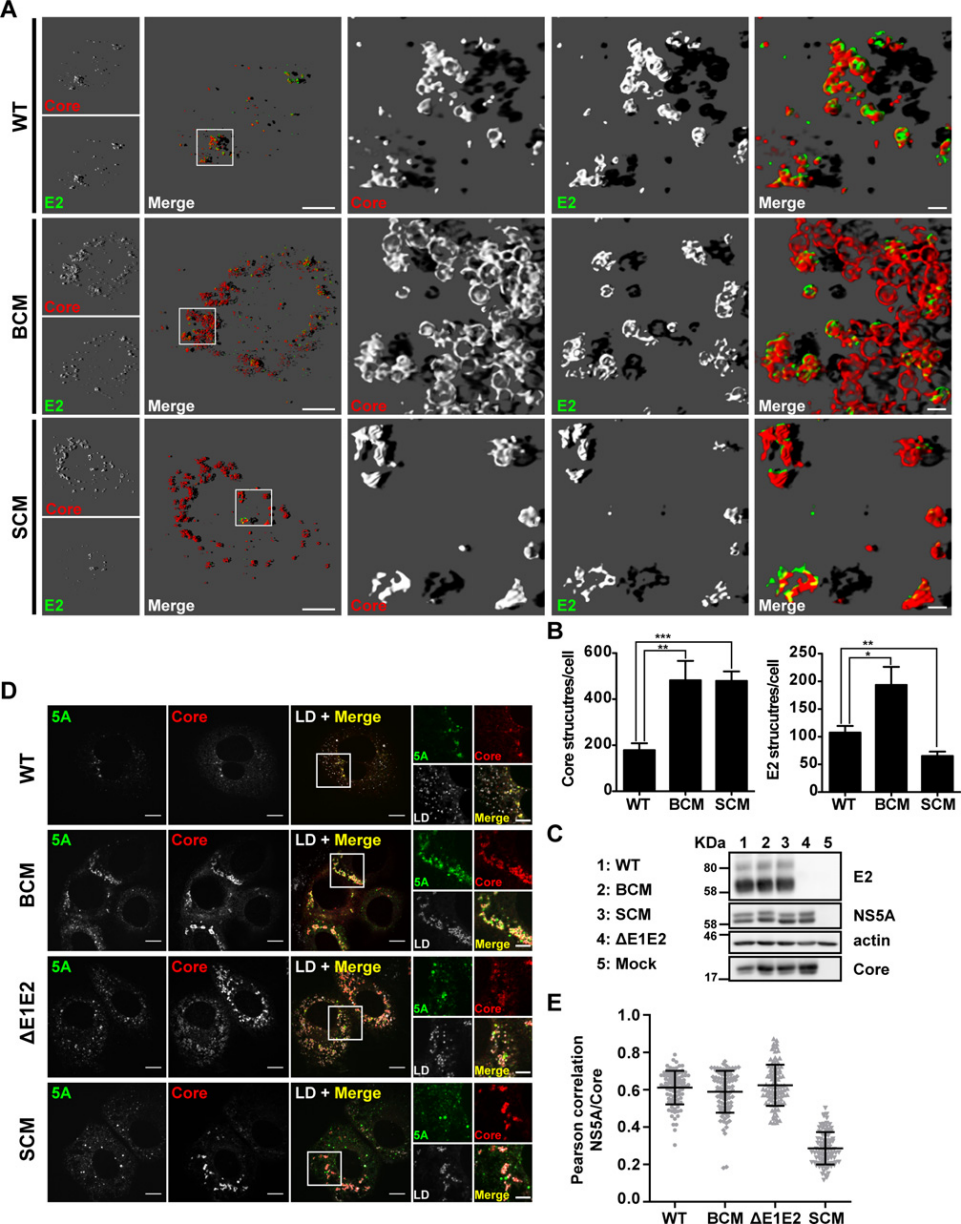
Mutations disrupting the NS5A basic cluster increase E2 accumulation in doughnut-like structures without affecting NS5A –core protein colocalization. Huh7-Lunet cells were transfected with the HCV genomes specified in the left and fixed 48 h later. HCV proteins were detected by immunofluorescence using mono-specific antibodies. (A) Representative images showing E2—core protein subcellular distribution. Images were generated with a spinning disk confocal microscope and deconvolved to generate a 3D reconstruction with deep projection (see Materials and Methods). The location of the cropped sections is indicated with white boxes in each overview panel. Scale bars represent 10 μm for overviews and 1 μm for cropped sections. (B) Quantification of core and E2 structures per cell. (C) Amounts of E2, NS5A and core protein were determined by Western blot using mono-specific antibodies; ß-actin served as loading control. (D) Representative images showing core—NS5A co-localization and their proximity to LDs (stained with BODIPY). Scale bars represent 10 μm for overviews and 5 μm for cropped sections. (E) Degree of NS5A - core co-localization as determined by Pearson correlation coefficient. Each dot represents one cell. For each HCV construct, the mean and SD of n = 100 is shown.

Since the impaired envelopment of HCV particles observed with the NS5A BC mutant might have been due to mislocalization of the structural proteins we determined their subcellular localization. Huh7-Lunet cells were transfected with Jc1 wildtype, the NS5A BC or the SC mutant, fixed 48 h later and E2 as well as core protein were detected by immunofluorescence. By using 3D reconstructions we found that in Jc1 wildtype-transfected cells the E2 protein was distributed in a dot-like pattern that overlapped with the core staining pattern (Fig 4A, upper row). Especially in case of the big dots, we often observed a doughnut-like shape, reminiscent of an accumulation around LDs [9]. In case of the NS5A BC mutant, the E2 and core staining patterns overlapped to a similar degree and we observed analogous doughnut-like structures (Fig 4A, middle row). However, we noted an increase of E2-containing structures, which correlated with a strong accumulation of core protein around LDs arguing that (i) recruitment of E2 to core-containing structures was not affected and (ii) core and E2 accumulated because of limited particle assembly and thus, limited release from the cell. As expected, in case of the NS5A SC mutant, core protein accumulated around LDs as well (Fig 4A bottom row); however E2-containing doughnut-like structures were much rarer as compared to the wildtype (Fig 4B). This was not due to differences of E2 amounts that were well comparable between wildtype and the NS5A mutants (Fig 4C) indicating an impaired recruitment of E2 to core protein-containing sites, which is consistent with an assembly defect prior to envelopment of virus particles [31].

We and others have earlier described that DIII of NS5A, specifically the serine cluster in its C-terminal region, is required for assembly of infectious HCV particles [30, 31, 40]. DIII of NS5A is necessary for colocalization with core around LDs and for NS5A –core interaction. Therefore, we evaluated if the NS5A BC mutation might affect the colocalization of these viral proteins. As shown in Fig 4D and 4E, this was not the case and only the NS5A SC mutation affected the colocalization of these viral proteins.

### NS5A lacking the basic cluster in DIII still interacts with core protein

**Fig 5:**
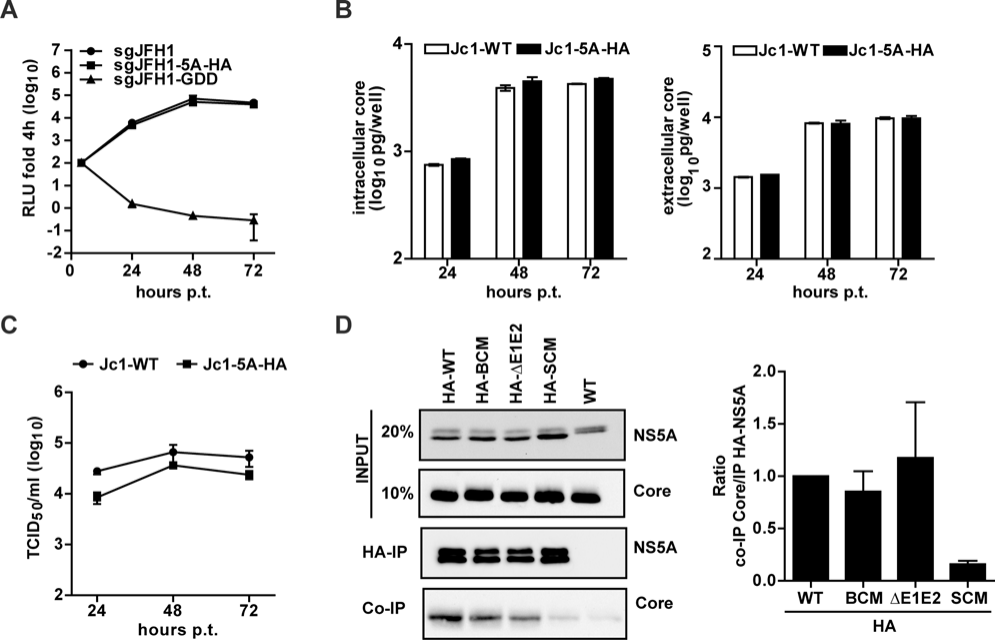
Loss of assembly competence of NS5A basic cluster mutant is not linked to altered NS5A - core interaction. (A) Replication kinetics of a subgenomic replicon containing a HA tag inserted at amino acid position 261 of NS5A Domain II (see Fig 1A). Huh7-Lunet cells were lysed at given time points after transfection and luciferase activity was determined. The replication-deficient mutant GDD served as negative control. (B) Quantification of core protein amounts contained in transfected cells or released into culture supernatants (left and right panels, respectively). Mean and SEM of three independent experiments is shown. (C) Kinetic of virus release from Huh7-Lunet cells transfected with full-length genomes specified on the top. Infectivity amounts were determined by limiting dilution assay. (D) NS5A - core interaction. Huh7-Lunet cells were transfected with HCV genomes specified on the top and 72 h later cells were lysed and NS5A was enriched by immunoprecipitation (IP) using a HA-specific monoclonal antibody covalently linked to agarose beads. Captured proteins were separated by electrophoresis into an 8% (NS5A) or 15% (Core) acrylamide gel and analyzed by Western blot using antibodies with specificities indicated in the right. Given amounts of input proteins were loaded onto the gel in parallel. The quantification shown in the right panel represents the enrichment of core protein that co-precipitated with HA-NS5A (mean and SEM of three independent experiments).

Having found that the NS5A BC mutation did not affect core—NS5A co-localization around LDs, we asked the question whether these proteins still interacted. To facilitate pull-down experiments, we inserted a HA-affinity tag into DII of NS5A at amino acid position 261, reported earlier to tolerate an insertion of 19 amino acid residues in the context of genotype 1b replicons [45]. The HA-tag, flanked by flexible linkers, was inserted into the subgenomic sgJFH1-Fluc reporter replicon (sgJFH1-5A-HA) to evaluate replication kinetics and into the full length Jc1 genome (Jc1-5A-HA) to determine virus particle production. *In vitro* transcripts derived from these constructs were transfected into Huh7-Lunet cells and RNA replication as well as particle production was analyzed as described above. As shown in Fig 5A, insertion of the HA-tag at this site of NS5A did not affect RNA replication kinetics. This result was corroborated by the comparable amounts of core protein accumulating in Jc1-5A-HA-transfected cells (Fig 5B, left panel). Moreover, for each time point core protein release from transfected cells was indistinguishable from the wildtype (Fig 5B, right panel), arguing that the HA-tag insertion did not interfere with virus particle assembly and release. Importantly, only a very moderate reduction of infectivity titer was found (3-fold reduction at 24 h and 2-fold reduction at 48 h and 72 h post transfection; Fig 5C). Thus, this HA-tagged NS5A variant was well suited to study NS5A interaction with core. Therefore, the tag was inserted into NS5A of the BC and the SC mutant as well as the Jc1-ΔE1E2 control construct. *In vitro* transcripts of these constructs were transfected into Huh7-Lunet cells and 72 h later cells were harvested and lysates were subjected to HA-specific immunoprecipitation (IP). The non-tagged Jc1 wildtype genome served as negative control. Immunocomplexes were resolved by SDS-PAGE and analyzed by Western blot. To determine the efficiency of NS5A –core co-precipitation, for each construct we calculated the inverse ratio of immunoprecipitated NS5A and co-precipitated core protein as quantified by densitometry. As shown in Fig 5D, the NS5A-HA-BC variant interacted with core protein with the same efficiency as the wildtype and the ΔE1E2 mutant. This was in stark contrast to the NS5A-HA-SC mutant reported earlier to no longer interact with core [31]. These results demonstrated that the NS5A BC is dispensable for interaction with the core protein.

### Requirement of the basic cluster in NS5A DIII for efficient RNA packaging

**Fig 6:**
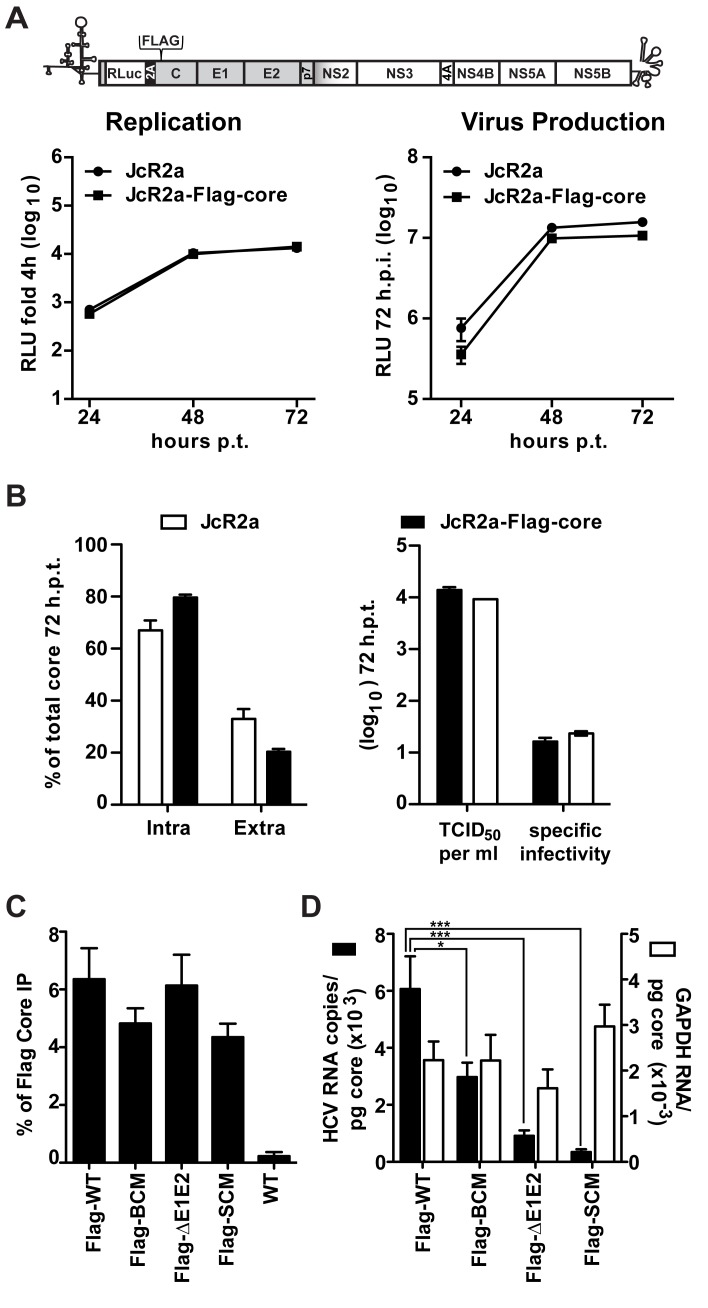
NS5A basic cluster mutant has a defect in core—HCV RNA association. (A) No impact of a Flag-tag inserted into core protein of a HCV reporter genome (JcR2a) on viral RNA replication and production of infectious virus particles. A schematic representation of the HCV genome indicating the insertion site in the core coding region (codon 2) is given in the top. Huh7-Lunet cells were transfected with the parental renilla luciferase reporter genome (JcR2a) or the variant containing a Flag-tag in core (JcRa2-Flag-core). Left panel: cells were lysed at given time points after transfection and replication was determined by measuring renilla luciferase activity. Right panel: culture supernatants of transfected cells harvested at given time points were used to infect naïve Huh7.5 cells and renilla luciferase activity was measured 72 h later. (B) Core protein amounts contained in transfected cells or released into culture supernatants 72 h after transfection are shown in the left panel (intra- and extracellular, respectively). Amounts of infectious virus released into culture supernatants and specific infectivity of released virus particles are given in the right panel. Mean and SEM of two independent experiments is shown. (C, D) Effect of mutations in NS5A on association of core protein with HCV RNA. Huh7-Lunet cells were transfected with HCV genomes specified in the bottom, 72 h later cells were lysed and Flag-tagged core was enriched by immunoprecipitation (IP) using a Flag-specific monoclonal antibody covalently linked to magnetic beads. (C) Efficiency of IP was determined by core-specific CMIA and normalized to the input. The non-tagged HCV genome (WT) served as technical control for specificity of immunoprecipitation. (D) HCV RNA and GAPDH mRNA (specificity control) coprecipitated with core protein was quantified by RT-qPCR and HCV RNA copy numbers (Y-axis in the left) or relative GAPDH mRNA values (Y-axis in the right) co-precipitated per pg core protein were calculated. Mean and SEM of ten independent experiments is shown. *, *p≤0*.05; ***, *p≤0*.*001*.

It is generally assumed that the RNA-binding protein NS5A is involved in the packaging of the genome into nucleocapsids, e.g. by facilitating the transfer of the viral RNA from the replication complex to the core protein at the assembly site (reviewed in [11, 19]). Therefore we determined a possible impact of the mutations disrupting the NS5A BC on RNA “loading” of core protein, which could explain diminished assembly. To allow efficient pull-down of core and measurement of co-precipitated HCV RNA, we inserted a Flag-tag into the core coding region of the monocistronic *Renilla* reporter virus JcR2a in order to avoid possible interference of the heterologous sequence with activity of the internal ribosome entry site (IRES) in the 5’ non-translated region (Fig 6A). In this construct the first 16 codons of the core coding region are duplicated upstream and downstream of the reporter gene, thus leaving the IRES unaltered [46]. Indeed, replication kinetics and particle production of this JcR2a-Flag-core mutant were similar to the parental JcR2a virus (Fig 6A, left and right panels, respectively). Moreover, amounts of intra and extracellular core, released infectivity and specific infectivity of released virus particles were comparable between the wildtype and the Flag-tag insertion mutant (Fig 6B, respectively) demonstrating that the Flag-tagged core protein was fully functional.

Taking advantage of this result, the mutations in NS5A were inserted into this JcR2a-Flag-core construct. *In vitro* transcripts of these genomes were transfected into Huh7-Lunet cells and 72 h later cell lysates were prepared. As a negative control for Flag-specific IP, the untagged Jc1 genome was analyzed in parallel. In order to avoid a co-precipitation of core—NS5A complexes, which would confound the quantification of core–RNA interaction, we first established lysis and IP conditions that disrupted core–NS5A interaction (S4 Fig). By using these conditions, we immunoprecipitated Flag-tagged core protein and measured its amounts by a core-specific CMIA whereas co-precipitated HCV RNA was quantified by RT-qPCR; in this assay we also quantified GAPDH mRNA contained in immunocomplexes to determine specificity. Efficiency of captured Flag-tagged core protein was similar between the Flag-tag constructs (Fig 6C). However, the percentage of co-precipitated HCV RNA differed significantly whereas no such differences were found with the GAPDH mRNA, demonstrating specificity of the immune capture (Fig 6D). After normalization to intracellular core protein and HCV RNA amounts we observed a 50% reduction of HCV RNA co-precipitating with the core protein in case of the NS5A BC mutant and a ~90% reduction in case of the SC mutant. Surprisingly, core–HCV RNA interaction was reduced ~80% in case of the ΔE1E2 mutant indicating that proper RNA packaging (i.e. formation of nucleocapsids) might be coupled to the budding process.

**Fig 7:**
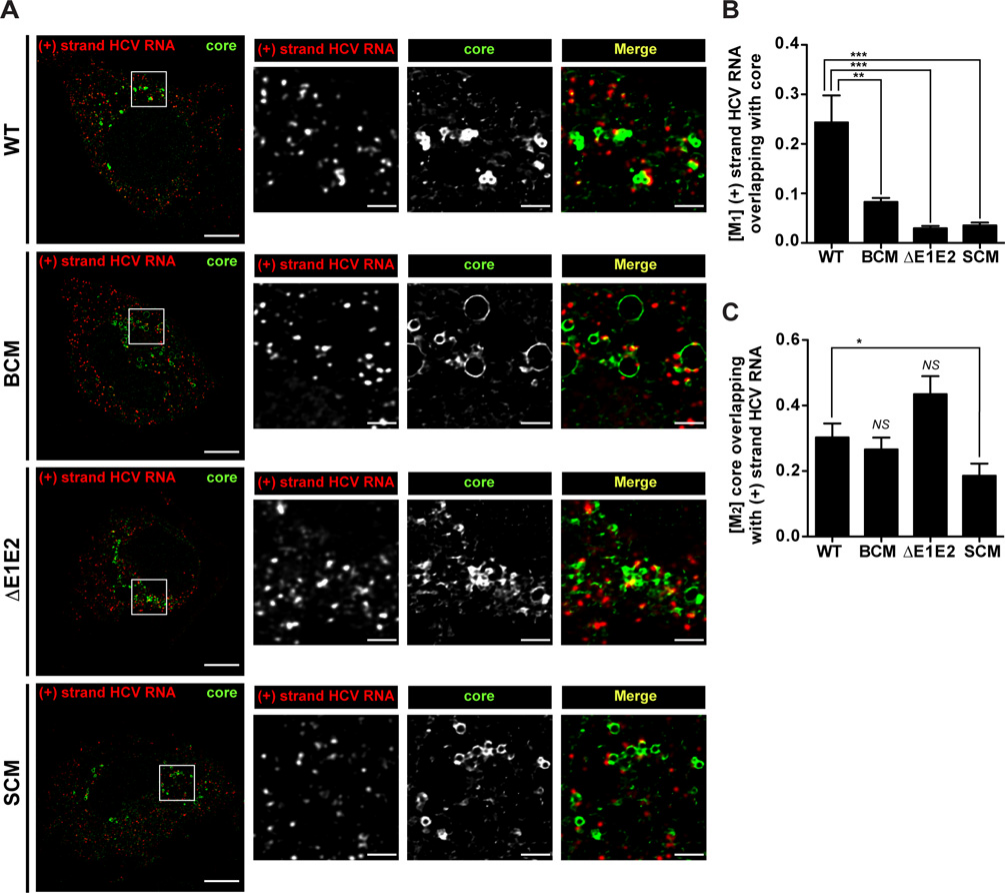
Impact of basic cluster mutations in NS5A on colocalization of HCV positive strand RNA with core protein. Huh7-hp cells were transfected with an HCV genome specified in the left of each row and fixed 72 h later. HCV positive strand RNA was detected by FISH using the Quantigene ViewRNA ISH Cell Assay (Affymetrix) and core protein was visualized by immunofluorescence using a mono-specific antibody. (A) Images were acquired with a spinning disk confocal microscope and deconvolved. The location of the cropped sections is indicated with white boxes in each overview panel. Scale bars represent 10 μm for overviews and 2 μm for cropped sections. (B) Mander’s overlap coefficients M_1_ for HCV positive strand RNA overlapping with core protein (red pixels overlapping with green pixels). (C) Mander’s overlap coefficients M_2_ for core protein overlapping with HCV positive strand RNA (green pixels overlapping with red pixels). For each HCV construct, the mean and SEM of n = 20 is shown. *, *p ≤0*.05; **, *p ≤0*.01; ***, *p ≤0*.*001; not significant (NS)*, *p ≥0*.*05*.

To corroborate our conclusions we determined the co-localization of positive strand HCV RNA and core protein by using fluorescence *in situ* hybridization (FISH) in combination with immunofluorescence. We used the Mander’s overlap coefficient (M) to quantify the fraction of HCV RNA that overlapped with core protein (M_1_) and the fraction of core protein overlapping with HCV RNA (M_2_). As shown in Fig 7, the fraction of positive strand RNA that co-localized with core protein (M_1_) was well detected in case of the wildtype; however in case of the NS5A BC mutant this co-localization was profoundly reduced. Likewise, HCV RNA–core co-localization was barely detected in case of envelope glycoprotein deletion mutant (ΔE1E2) and the NS5A SC mutant. On the contrary, when comparing the fraction of core protein that co-localized with positive strand HCV RNA (M_2_; Fig 7C), only the NS5A SC mutant showed a significant reduction, consistent with the assumption that this mutation has a defect in recruiting (RNA containing) viral replicase to the core protein [31]. In conclusion, these results suggest that the assembly defect of the NS5A BC mutant might not be due to impaired recruitment of core protein to sites containing viral RNA, but rather to a defect of the subsequent step, i.e. formation of RNA-containing nucleocapsids.

### Impaired association of the NS5A BC mutant with HCV RNA

**Fig 8:**
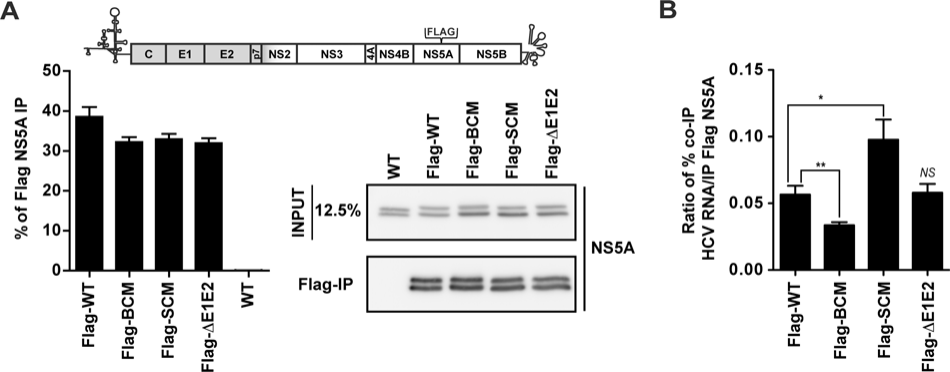
Mutations affecting the NS5A basic cluster reduce interaction with HCV RNA. (A) Schematic representation of the Jc1 genome containing a Flag-tag in NS5A DII. Huh7-Lunet cells were transfected with HCV genomes specified in the bottom and 72 h later cells were lysed and NS5A was enriched by immunoprecipitation (IP) using a Flag-specific monoclonal antibody covalently linked to magnetic beads. Captured NS5A proteins were separated by electrophoresis into an 8% acrylamide gel and analyzed by Western blot using a mono-specific NS5A antibody. The same amounts of input proteins were loaded onto the gel in parallel. The efficiency of the Flag-NS5A IP was determined by quantifying the bands and normalizing to the input signals. The non-tagged HCV genome (WT) served as technical control for specificity of immunoprecipitation. (B) Quantification of HCV RNA co-precipitated with Flag-NS5A. HCV RNA and GAPDH mRNA (specificity control) was determined by RT-qPCR. The percentage of viral RNA copies co-precipitated (co-IP) with NS5A was determined and used to calculate the enrichment of HCV RNA co-precipitated with NS5A. Note that GAPDH mRNA was below the detection limit in the co-precipitated samples and therefore is not displayed. Mean and SEM of six independent experiments is shown. *, *p ≤0*.05; **, *p ≤0*.*01; not significant (NS)*, *p ≥0*.*05*.

It has been reported that all domains of NS5A interact with HCV RNA [38, 39]. Therefore, we determined a possible impact of the basic cluster in DIII on association with the viral RNA genome by using NS5A-specific IP. For technical reasons we replaced the HA tag at amino acid position 261 of NS5A by a Flag tag, because of high non-specific binding of HCV RNA to HA antibody-coupled agarose beads. RNA replication and virus particle production of this Jc1 variant was similar to the wildtype, thus proving full functionality of this construct (S5 Fig). Next we inserted the NS5A mutations into this Jc1-derived genome and *in vitro* transcripts were transfected into Huh7-Lunet cells. After 72 h cell lysates were prepared using conditions that disrupt the NS5A - core protein interaction. The untagged Jc1 genome was analyzed in parallel as (technical) negative control. Amounts of immune-captured Flag-tagged NS5A proteins were quantified by Western blot and co-precipitated HCV RNA and GAPDH mRNA was quantified by RT-qPCR. As shown in Fig 8A, the capture efficiency of Flag-tagged NS5A was comparable between the constructs. When we quantified the relative amount of viral RNA coprecipitating with NS5A of the BC mutant, we observed a 45% reduction whereas in case of the envelope deletion mutant (ΔE1E2) no difference to the wildtype was found (Fig 8B). This result suggests that the defect in core—HCV RNA association and envelopment observed with the NS5A BC mutant might be indirect and result from the insufficient RNA amounts delivered by NS5A to core protein. In contrast, in case of the NS5A serine cluster mutant we detected a ~70% increase of viral RNA coprecipitating with NS5A (Fig 8B) arguing that this mutation might primarily affect RNA delivery from NS5A to core protein as a result of impaired core–NS5A interaction. In all immunocomplexes capture of HCV RNA was specific, because GAPDH mRNA amounts in the immunocomplexes were below the detection limit. Taken together these results reveal two elements in DIII of NS5A required for distinct, but closely coupled steps of assembly: RNA binding to NS5A and subsequent transfer to the core protein (BC) and interaction of NS5A with core protein to allow RNA transfer (SC).

## Discussion

Assembly of HCV particles requires a spatio-temporally coordinated association of the replicase, notably the NS3 helicase and NS5A, with the core protein to allow packaging of the RNA genome into the virion [11, 19]. We and others have earlier shown that NS5A plays a decisive role in this process. First, it interacts with core protein via NS5A DIII [30, 31]; second, NS5A is recruited to cLDs where core protein accumulates [9, 30, 31]; third, assembly requires the phosphorylation of a serine residue in NS5A DIII by casein kinase IIα [40]; fourth, NS5A facilitates the association of core protein with the viral RNA genome ([31] and this report); fifth, NS5A interacts with the p7-NS2 complex that is required for the envelopment of the HCV particles [47]; sixth, NS5A also interacts with apolipoprotein E that is incorporated into the virion [18, 48–52] and with Annexin A2, which is a host cell membrane sorting protein enhancing HCV assembly [53].

In the present study we extended this list by providing evidence that RNA binding to NS5A is important for an early step in the assembly pathway of infectious HCV particles. This regulatory function is linked to a cluster of four basic amino acid residues at the very N-terminus of DIII, whose positive charge is conserved across all HCV genotypes. A quadruple mutation affecting all basic residues of this region did not affect HCV RNA replication, but had numerous effects on the production of infectious HCV particles:

- Reduced titers of infectious extra- and intracellular virus particles;
- Reduced core protein release;
- Massively reduced specific infectivity of extra- and intracellular virus particles;
- Impaired formation of nucleocapsids and envelopment of virions;
- Diminished colocalization of positive strand HCV RNA with core protein;
- Reduced core–HCV RNA association;
- Reduced NS5A –HCV RNA association.

**Fig 9:**
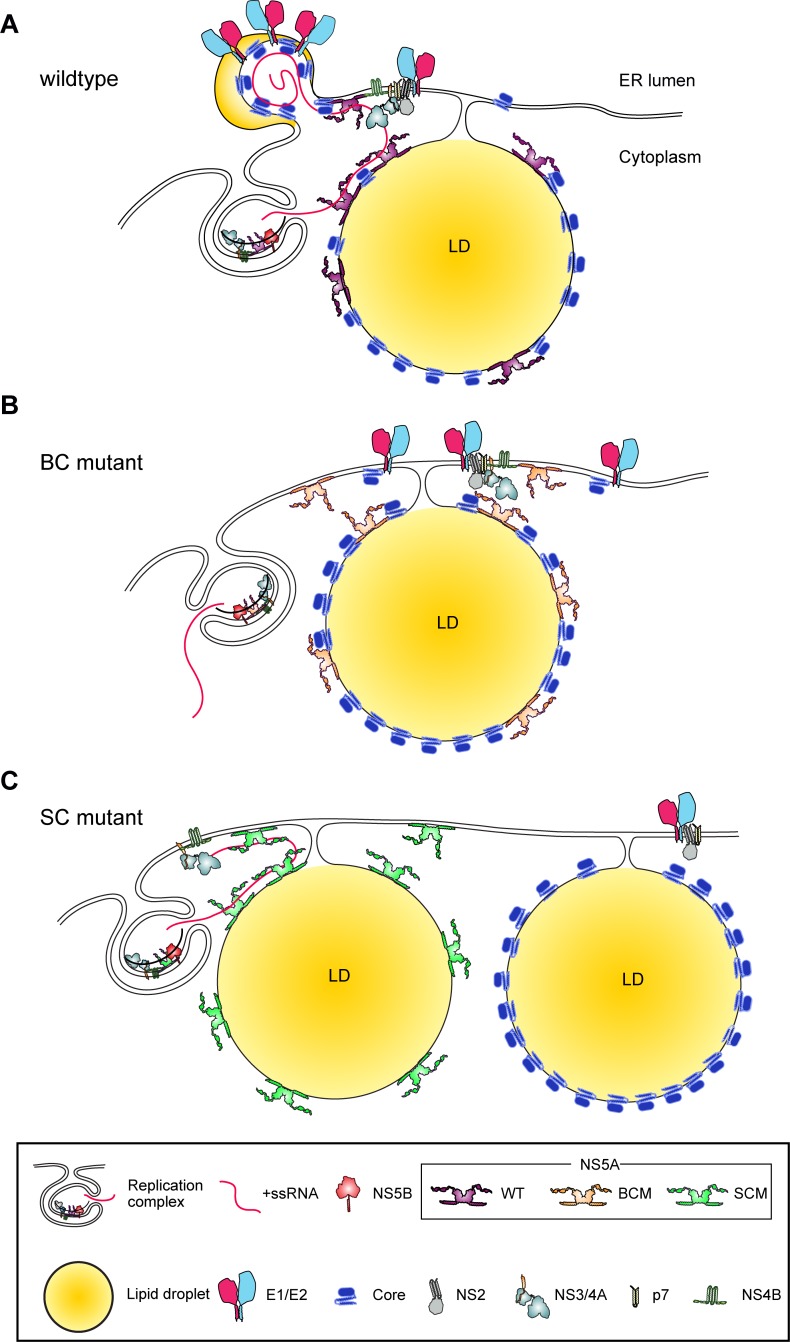
Hypothetical roles of serine- and basic clusters in NS5A domain III for the assembly of infectious HCV particles. (A) NS5A interacts with core protein to recruit (RNA-containing) replication complexes (RCs) to HCV assembly sites where Core, E1, E2, p7 and NS2 reside. NS5A delivers the viral genome, eventually in conjunction with NS3 [78], from the RCs to core protein to trigger genome encapsidation and nucleocapsid formation. This induces their budding into the ER lumen that might be promoted by the envelope glycoproteins E1 and E2 in conjunction with p7 and NS2 [11, 19]. (B) The NS5A basic cluster (BC) mutant is able to recruit the RCs to the assembly site by interacting with core protein, but is unable to associate with viral RNA for the assembly process. (C) The NS5A serine cluster (SC) mutant does not affect NS5A-HCV RNA association, but fails to interact with core protein and therefore the RCs are not recruited to the assembly site. As a result NS5A and core protein accumulate around distinct cytosolic lipid droplets (cLDs). Overall, both NS5A mutants are unable to load core protein with viral RNA, although for different reasons, and as a consequence envelopment of nucleocapsids is impaired.

The observed impairment of NS5A –RNA interaction is consistent with a previous study showing that all NS5A domains bind to HCV RNA, albeit with different affinities [39]. Since core–NS5A interaction was not affected by the BC mutation, we propose that the reduced RNA binding to NS5A impairs the delivery of the RNA genome to the core protein (Fig 9). According to this model, the association of core protein with viral RNA would be required for nucleocapsid assembly, which was impaired (indirectly) by the mutation affecting the BC in NS5A. Moreover, our data provide strong evidence that nucleocapsid formation and envelopment are tightly coupled as deduced from the proteinase K sensitivity of core protein in cells containing the BC mutant and the absence of fast sedimenting core protein complexes. Interestingly, the analogous observation was made with the SC mutant that was also very much impaired in nucleocapsid formation as well as particle envelopment. However, the main difference is that the SC is not required for association with viral RNA, but instead for interaction with the core protein as deduced from our co-precipitation studies (Figs 5D and 8B, respectively). Thus, according to our model, both mutants would be unable to deliver viral RNA to the core protein, but for different reasons (Fig 9B and 9C): mutations affecting the BC would reduce RNA binding to NS5A, and subsequently to core during the assembly reaction, whereas NS5A lacking the SC in DIII associates with viral RNA, but owing to the loss of interaction with core protein is unable to deliver it. Another difference arising from the mutations in NS5A relates to the subcellular localization of the structural proteins and NS5A. We found that the BC mutant supported recruitment of E2 to core-containing structures as well as core–NS5A colocalization around cLDs. In fact, E2-positive (ring-like) core structures were more abundant in BC mutant than in wildtype virus containing cells, which is probably due to the accumulation of these proteins at assembly sites as a result of the inability to form proper nucleocapsids. In contrast, in case of the SC mutant NS5A recruitment of E2 to core-containing structures was reduced and core only rarely colocalized with NS5A around cLDs. This result fits well to our earlier observation made with a deletion mutant lacking most of NS5A DIII [30] and raises the question how the co-recruitment of core and NS5A to the same cLD might be mediated. Obviously it is not linked to NS5A –RNA interaction, because this is unaltered with the SC mutant, but rather to core–NS5A interaction. Whether this interaction is direct or mediated by cellular factors such as VAP-A/B [50, 54] that are involved in intracellular trafficking remains to be determined.

Although the BC mutation primarily affected assembly of infectious HCV particles, we note that virus particles were still released. However, their specific infectivity (i.e. the number of infectivity units per core protein amount) was massively reduced arguing that these particles were not properly assembled or matured. Several possibilities could account for this defect such as impaired recruitment of host cell factors involved in the maturation of the virion. Alternatively, reduced specific infectivity of the BCM might result from a modified virus particle composition such as altered lipid profile or content. Further studies will be required to address these possibilities.

Taking advantage of a HCV genome encoding a fully functional Flag-tagged core protein, we observed that core—HCV RNA interaction was reduced by ~50% in case of the NS5A BC mutant and ~90% in case of the NS5A SC mutant, the latter being in agreement with previous findings [31]. Surprisingly, this interaction was also reduced massively (~80%) in case of the envelope glycoprotein deletion mutant (ΔE1E2), further supporting the notion that nucleocapsid formation, which requires RNA–core interaction, and envelopment are coupled. One can assume that the interaction of core with viral RNA drives core protein oligomerization that -via its domain 2- is bound to the ER membrane at the assembly site (Fig 9A). The successive oligomerization would push the assembling capsids through the ER membrane, thus acquiring the viral envelope. It is unclear how this process is linked to the acquisition of the envelope glycoproteins but this might be facilitated by cellular lipoproteins that become an integral part of the virus particle such as apolipoprotein A, C or E [18, 55–58]. In addition, it has been reported that core protein interacts with E1 [59–61] and this interaction appears to be mediated by residues located in-between two hydrophobic regions of E1 [61]. Although E1 and E2 have a type I membrane topology, for E1 a polytopic membrane topology has been proposed to coexist [61, 62] with the loop between the two hydrophobic regions being exposed towards the cytosol to form a cytosolic loop that could interact with core protein. Moreover, it was shown that core mutants that do not oligomerize *in vitro* are unable to interact with E1 [61]. While core–E1 association is an attractive possibility how HCV acquires its envelope, these observations need to be confirmed in an infection system and it will be interesting to determine whether E1 mutations affecting core binding affect core–HCV RNA association. Along the same lines, it was shown that E1 and E2 heterodimers present in secreted HCV particles form disulfide bridges and it has been proposed that the E1/E2 ectodomains promote budding by lateral interactions thereby “pulling” virus particles towards the ER lumen [63]. It will be interesting to investigate if this heterodimerization of the ectodomains also contributes to RNA loading of core protein.

In summary, we provide compelling evidence that NS5A DIII contains two regions that regulate the assembly process at two distinct steps. Recently, directly acting antiviral drugs targeting NS5A have been developed inhibiting both HCV RNA replication and the production of infectious HCV particles [64–66]. While replication inhibition could be ascribed, at least in part, to the inhibition of the biogenesis of the membranous HCV replication factory (the membranous web), it is unclear how this inhibitor class blocks virus production. Taking advantage of the observations made in the present study and the novel tools, it will be interesting to investigate, amongst others, the possible impact of these inhibitors on (NS5A-dependent) core protein loading with viral RNA. Obviously, the tools and methods described here will help to solve the still mysterious mechanism by which this exceptionally antiviral potency is brought about.

## Materials and Methods

### Plasmids

The constructs pFK-Jc1-δg (Jc1), pFK-Jc1ΔE1E2-δg (Jc1ΔE1E2), pFK-JcR2a-δg (JcR2a), pFK-Jc1-Flag-E2-δg (Jc1E2^FLAG^), pFK-i389LucNS3-3’-JFH1-δg (sgJFH1-FLuc-WT) and pFK-i389LucNS3-3’-NS5BΔGDD-JFH1-δg (sgJFH1-FLuc-GDD) have been described previously [4, 18, 30, 46, 67]. Nucleotide and amino acid numbers refer to the genome of the JFH1 isolate (GenBank accession no. AB047639). The coding sequence for the Flag- (DYKDDDDK) or HA- (YPYDVPDYA) epitope tag was inserted in-frame after codon 261 into the NS5A coding region giving rise to plasmids pFK-i389LucNS3-3’-JFH1-NS5A-HA-δg, pFK-Jc1-NS5A-HA-δg, pFK-Jc1ΔE1E2-NS5A-HA-δg, pFK-Jc1-NS5A-Flag-δg and pFK-Jc1ΔE1E2-NS5A-Flag-δg. Plasmid pFK-JcR2a-Flag-Core-δg was generated by inserting the Flag tag coding sequence in-frame after the second codon of the core coding region of plasmid pFK-JcR2a-δg as described previously [68]. Plasmid pFK-JcR2aΔE1E2-Flag-Core-δg lacking the coding region of the envelope glycoproteins (amino acid residues 218–567) were obtained by transferring a *Cla*I—*Kpn*I DNA fragment isolated from pFK-Jc1ΔE1E2-δg into pFK-JcR2a-Flag-Core-δg. NS5A mutations (R352E, R353E, R354E, R355E, R352-353E, R352-354E, R353-355E, R352-355E, R352-355A and S452/454/457A) were created by PCR-based site-directed mutagenesis and DNA fragments were inserted into the parental plasmids described in the text. The cDNA clone pCV-H77c of the genotype 1a strain H77 (kindly provided by Jens Bukh, University of Copenhagen, Denmark) has been described previously [69] and was used to insert a combination of five cell culture adaptive mutations enhancing RNA replication [41] and virus production in Huh7 cells [16]. Nucleotide and amino acid numbers refer to this H77 isolate (GenBank accession no. AF011751; [69]). The five adaptive mutations (NS3 [Q1067R, V1651I], NS4A [K1691R] and NS5A [K2040R, S2204I]) were generated by PCR-based site-directed mutagenesis and inserted into the H77c genome, thus creating the H77S variant [41]. The NS5A quadruple mutation R356E, K357-358E and R359E was generated by PCR-based site-directed mutagenesis and inserted via a *Sna*BI-*Hind*III DNA fragment into the H77S cDNA clone. Integrity of all constructs was confirmed by nucleotide sequence analysis. Detailed information about DNA cloning is available upon request.

### Antibodies

The cellular proteins β-actin and ADRP (Adipose differentiation-related protein) were detected using mouse monoclonal antibody A5441 from Sigma-Aldrich (Munich, Germany) and sc-377429 Santa Cruz (Heidelberg, Germany), respectively. Viral proteins were detected with the following antibodies: mouse monoclonal antibody raised against NS3 of the JFH1 isolate (NS3-2E3; generated in cooperation with H. Tang, Florida State University, USA); mouse monoclonal antibody 9E10 raised against NS5A of the Con1 isolate (kind gift of C. M. Rice, Rockefeller University, New York, USA); mouse monoclonal antibody recognizing core protein (C7/50, sc-57800, Santa Cruz, TX, USA); rabbit polyclonal antibody reacting with core (C830) [70]; mouse and rat monoclonal antibodies recognizing E2, AP33 (a kind gift from Genentech Inc., CA, USA) [71] and 3/11 (kind gift from J.A. McKeating, Birmingham, UK), respectively. Anti-HA antibodies coupled to agarose beads, anti-Flag M2 magnetic beads and secondary horseradish peroxidase-conjugated antibodies were purchased from Sigma-Aldrich.

### Cell culture, electroporation of HCV in vitro transcripts and transient replication assay

Human hepatoma cell lines Huh7-Lunet [72], Huh7-hp [73] and Huh7.5 [74], which are highly permissive for HCV RNA replication were used for electroporation and infection assays. Cell monolayers were grown in Dulbecco’s Modified Eagle Medium (DMEM), supplemented with 2 mM L-glutamine, nonessential amino acids, 100 U/ml penicillin, 100 μg/ml streptomycin and 10% fetal calf serum (complete DMEM). Transient HCV RNA replication assays were performed as described previously [69, 75], In brief, plasmid DNA was linearized with *Mlu*I or *Xba*I and used for *in vitro* transcription. Five to 10 μg of in vitro transcripts were used for transfection by electroporation of 4 x 10^6^ Huh7-Lunet cells. Depending on the amount of transfected cells needed for a given experiment one or several electroporations were performed, cells were pooled in complete DMEM and seeded into cell culture dishes as required. Replication was determined by measuring luciferase activity in case of genomes containing the *Firefly* or *Renilla* luciferase reporter gene at serial time points post transfection. Replication kinetics were calculated by normalizing the relative light unit (RLU) values measured at different time points after transfection to the respective 4 h value, reflecting transfection efficiency.

### Infectivity assays

To determine the amount of infectious extracellular virus, supernatants from transfected cells harvested at various time points after transfection were filtered through a 0.45 μm-pore size filter. In case of luciferase reporter viruses infectivity was determined as described elsewhere [15]. In brief, 5 x 10^4^ Huh7.5 cells were seeded into a 24-well plate one day prior to infection. Duplicates of cells were inoculated with supernatants for 4 h, medium was replaced by complete DMEM and 72 h later cells were harvested to measure luciferase activity in cell lysates. Infectivity titers of non-reporter gene HCV variants and in some cases also of reporter virus genomes were determined by using a limiting dilution assay [14]. In brief, 1 x 10^4^ Huh7.5 cells were seeded into a 96-well plate one day prior infection; cells were infected in sixtuplicates for 72 h and fixed with ice-cold methanol. Infected cells were detected by using the NS3-specific 2E3 mouse monoclonal antibody (dilution 1:1000). In case of infectious virus produced with the H77S isolate, infected cells were detected in the same manner, but using a combination of the monoclonal antibodies NS5A-9E10 and core C7/50 (dilution 1:2000 and 1:200, respectively). Primary antibodies were detected with a peroxidase-conjugated goat anti-mouse polyclonal secondary antibody (Sigma) at a dilution of 1:200. The tissue culture 50% infectivity dose (TCID_50_) was determined as described elsewhere [4] and the formula provided at http://www.klinikum.uni-heidelberg.de/Downloads.126386.0.html?&L=0. To determine intracellular infectivity titers, transfected cells were disrupted by several freeze–thaw cycles as described earlier [76]. In brief, transfected Huh7-Lunet cells were extensively washed with PBS, scraped off the plate into PBS and centrifuged for 5 min at 700 × g. Cell pellets were resuspended in complete DMEM and subjected to three cycles of freezing and thawing by using liquid nitrogen and a thermo block set to 37°C. Cell debris was removed by centrifugation at 20,000 × g for 10 min at 4°C. Culture supernatants from transfected cells were treated in the same way and infectivity was determined in parallel by limiting dilution assay as described above.

### Immunoprecipitation

All steps were carried out at 4°C. Transfected cells grown in 15 cm-diameter dishes were washed twice with cold PBS, scraped off the plate in PBS and centrifuged for 5 min at 700 × g. For Core-Flag and NS5A-Flag specific immunoprecipitations, cell pellets were resuspended in 1ml lysis buffer 2 (10 mM Tris-HCl [pH 7.5], 150 mM NaCl, 10 mM NaF, 1 mM Na_3_VO_4_, 0.5% Triton X-100). In case of NS5A-HA specific immunoprecipitations, cell pellets were resuspended in buffer 1 (20 mM Tris-HCl [pH 7.5], 50 mM NaF, 10 mM Na_3_VO_4_ and 0.1% NP-40). Both buffers were supplemented with a cocktail of protease inhibitors (Complete EDTA free; Roche). The cell lysate was incubated for 1 h on ice and precleared by centrifugation at 20,000 × g for 1 h. Supernatants were incubated for 4 h with either Flag-magnetic or HA-agarose beads in an inversion mixer at 4°C and beads were washed thereafter 3–5 times with lysis buffer. In case of immunoprecipitation with Flag beads the last wash was performed with TBS buffer (50 mM Tris-HCl [pH 7.5] and 150 mM NaCl). Bound proteins were eluted in 3% SDS-TBS buffer whereas RNA was extracted by using the NucleoSpin RNAII kit (Macherey-Nagel). Captured RNA was quantified by using TaqMan RT-qPCR. For HCV RNA bound to core or to NS5A, a primer set specific for the 5’NTR or 3’NTR was used, respectively.

### Quantification of HCV core protein

Transfected cells were harvested in 0.5% Triton X-100 in PBS supplemented with protease inhibitors. Lysed samples were cleared by centrifugation at 20,000 × g for 10 min at 4°C. Precleared samples and cell culture supernatants were diluted to a final concentration of 0.5% Triton X-100 in PBS. Core protein amounts were determined by using a commercial chemiluminescent microparticle immunoassay (CMIA) according to the instructions of the manufacturer (6L47, ARCHITECT HCV Ag Reagent Kit, Abbott Diagnostics, USA). Since core protein amounts detected 4 h after transfection are derived from translation of input RNA, values obtained for this time point were used to normalize for transfection efficiency.

### Western blot analysis

Samples were mixed with 5X Laemmli buffer and incubated for 5 min at 95°C. Proteins were separated by electrophoresis into 8% (for NS5A, E2, β-actin and ADRP) or 15% (for core protein) polyacrylamide gels and transferred to a PVDF membrane. Membranes were blocked with PBS containing 0.5% Tween-20 (PBST) supplemented with 3% bovine serum albumin (BSA; Applichem). Primary antibodies [NS5A (1:10,000), Core (C7/50; 1:1,000), ADRP (1:1,000), β-actin (1:20,000) and E2 (3/11; 1:500)] were diluted in PBST containing 1% BSA and membranes were incubated with the antibodies for 1 h at RT or overnight at 4°C. Membranes were washed 3 times with PBST and incubated for 1 h at RT with the corresponding horseradish peroxidase-conjugated secondary antibody. After 3 washes with PBST, bound secondary antibody was detected by using the Western lightning plus-ECL reagent according to the instructions of the manufacturer (PerkinElmer, Waltham, MA). Signals were detected by using the Intas ChemoCam Imager 3.2 (Intas, Göttingen, Germany) and quantified with the LabImage 1D Intas software package.

### Quantitative detection of HCV RNA by RT-qPCR

Total RNA prepared from transfected cells or magnetic beads was eluted from NucleoSpin RNAII columns in a volume of 50 μl RNase-free water. Five microliters of the respective sample were used for RT—quantitative PCR analysis using a BioRad CFX96^TM^ Real-Time System/C1000 Thermal cycler. Amplifications were conducted in triplicate with the One Step RT-PCR Kit using the following primers and 39-phosphate-blocked, 6-carboxyfluorescine (6FAM)- and tetrachloro-6-carboxyfluorescine (TAMRA)- labeled probes for HCV (TIB Molbiol, Berlin, Germany) and VIC- and TAMRA- labeled probe for glyceraldehyde 3-phosphate dehydrogenase (GAPDH, Applied Biosystems): HCV-JFH1 5’NTR Taqman probe A195: 5’-6FAM-AAA GGA CCC AGT CTT CCC GGC AAT T-TAMRA-3’; HCVJFH1-S147: 5’-TCT GCG GAA CCG GTG AGT A-3’; HCVJFH1-A221: 5’-GGG CAT AGA GTG GGT TTA TCC A-3’; HCV-JFH1 3’NTR Taqman probe A9592: 5’-6FAM-CTA CTT TCT TTC TTG GTG GCT CCA TC-TAMRA-3’; HCVJFH1-S9539: 5’-TCC CTC TTT CTT CCC TTC TCA TC-TAMRA-3’; HCVJFH1-9613: 5’-GCT AGC CGT GAC TAG GGC-3’; GAPDH Taqman probe: 5’-VIC-CAA GCT TCC CGT TCT CAG CCT- TAMRA; GAPDH-S: 5’-GAA GGT GAA GGT CGG AGT C-3’; GAPDH-A: 5’-GAA GAT GGT GAT GGG ATT TC-3’. The total reaction volume was 15 μl and contained the following components: 4 mM MgCl_2_, 0.66 mM deoxynucleoside triphosphates (dNTPs), 0.266 μM (each) HCV and GAPDH probe, 1 μM of each sense and antisense HCV and GAPDH primer and 0.6 μl of enzyme mix. The amounts of HCV RNA were calculated by comparison to serially diluted *in vitro* transcripts included in parallel in the RT-qPCR analysis. Reactions were carried out in three stages with the following conditions: stage 1, 60 min at 50°C for reverse transcription reaction; stage 2, heat inactivation of reverse transcriptase and activation of *Taq* polymerase for 15 min at 95°C; stage 3, 40 cycles, with 1 cycle consisting of 15 sec at 95°C and 1 min at 60°C.

### Rate zonal centrifugation and proteinase K protection assay

These assays were performed as described previously with a few minor modifications [42, 44]. In brief, transfected cells seeded into a 10 cm-diameter dish were harvested 12, 24 and 48 hours later. Cells were washed with ice-cold PBS, scraped off the plate into PBS and centrifuged for 5 min at 700 × g at 4°C. Cell pellets were resuspended in 250 μl TNE buffer (10 mM Tris-HCl [pH 8], 150 mM NaCl and 2 mM EDTA) and subjected to five freeze and thaw cycles. Cell debris was removed by centrifugation at 1,700 × g for 5 min at 4°C. Pre-cleared samples were loaded on top of a continuous 0–30% sucrose/TNE gradient and centrifuged at 270,000 × g for 1 h at 4°C in a SW 40 Ti rotor (Beckman Coulter, Inc.). Nine fractions were collected from the top of the gradient and their densities were calculated by measuring their refractive index. For proteinase K (PK) protection assay cells were harvested 48 h post transfection as described above and precleared samples were divided into three equal aliquots that were left untreated or incubated with 15 ng of PK (P2308, Sigma) in the absence or presence of 1% Triton X-100 for 40 min on ice. PK was inactivated by adding 10 mM phenylmethylsulfonyl fluoride (AppliChem) to the reaction that was incubated for 10 min on ice and subsequently heated for 5 min at 95°C. Samples were analyzed by Western blot and core protein amounts were determined by CMIA.

### Ultracentrifugation

Supernatants and transfected cells were harvested 48 h post transfection. Supernatants were filtered through a 0.45 μm-pore size filter and concentrated by centrifugation using an Amicon ultra column (100K NMWL; Millipore, Germany) according to the instructions of the manufacturer. Concentrated supernatant was layered on top of a discontinuous Optiprep (Axis-Shield) gradient formed by steps of 20, 40, 60 and 80% Optiprep/TNE (v/v). To analyze intracellular virus particles, transfected cells were washed with ice-cold PBS, scraped off the plate into PBS and centrifuged for 5 min at 700 × g at 4°C. Cell pellets were resuspended in 500 μl TNE buffer and subjected to five freeze and thaw cycles. Cell debris was removed by centrifugation at 1,700 × g for 5 min at 4°C. Pre-cleared samples were loaded on top of a discontinuous gradient formed by steps of 20, 30, 40, 50, and 60% Optiprep/TNE (v/v). Gradients were centrifuged for 16 h at 120,000 × g in a SW60Ti rotor (Beckmann) at 4°C. Fractions were taken from the top of the gradient and their density, infectivity titer and core protein amounts were determined as described above.

### Affinity purification of E2^FLAG^ HCV particles

This assay was performed as described previously [18] with a few minor modifications. In brief, supernatants of transfected Huh7-Lunet cells were harvested after 48 h, filtered through a 0.45 μm pore size filter and concentrated by centrifugation using an Amicon ultra column (100K NMWL; Millipore, Germany) according to the instructions of the manufacturer. One milliliter of concentrated supernatant was incubated overnight with 50 μl bed volume of Flag magnetic beads in an inversion mixer at 4°C. Magnetic beads were washed five times with 1 ml PBS (20-fold excess of bead volume) and captured virions were eluted in two cycles by using 5 bed volumes of TNE buffer containing 100 μg/ml FLAG peptide (Sigma) for each cycle. Eluates were pooled and infectivity titers, core and HCV RNA amounts were determined as described above.

### Immunofluorescence analyses and image deconvolution

Transfected Huh7-Lunet cells were seeded onto glass coverslips that had been placed into 24 well-plates. Forty-eight hours after electroporation, cells were washed with PBS and fixed with 4% paraformaldehyde for 20 min at RT. Fixed cells were permeabilized for 5 min at RT with either digitonin (50 μg/ml in PBS) in case of NS5A, core and lipid droplets (LDs) co-staining or with 0.5% Triton X-100 in PBS in case of E2 and core co-staining. Permeabilized cells were washed with PBS and blocked with PBS containing 5% (w/v) BSA (Sigma) for 30 min at RT. NS5A and E2 were detected by using the mouse monoclonal antibodies 9E10 (dilution 1:1,000) and AP33 (dilution 1:300), respectively. Core was detected by using the monospecific rabbit polyclonal antiserum C-830 (dilution 1:200). After 1 h incubation at RT, cells were washed three times with PBS and incubated for 1 h in the dark with a 1:1,000 dilution of Alexa 488, 564 or 647-conjugated secondary antibody (Invitrogen, Molecular Probes). Antibodies were diluted in PBS– 1% BSA. LDs were stained with 20 μg/ml BODIPY493/503 (Invitrogen, Molecular Probes) during secondary antibody incubation. Cells were washed once with PBS and nuclei were stained with a 1:5,000 diluted solution (in PBS) of DAPI (Sigma-Aldrich) for 1 min. After three times washing for 10 min each with PBS, cells were mounted on glass slides with Vectashield (Vector Laboratories Inc.). Cells were imaged on an Ultraview ERS spinning disk (PerkinElmer Life Sciences) mounted on a Nikon TE2000-E inverted confocal microscope equipped with a Plan-Apochromat VC 100X lens (NA 1.4). Channels were recorded sequentially onto a Hamamatsu Orca Flash 4 camera by using an emission discrimination option in the following order: 647/700, 568/610, 488/510, 405/440 (emission/excitation). For deconvolution and 3D reconstruction, optical slices were acquired at 0.125-μm Z spacing. Pearson’s correlation coefficient (R_r_) of NS5A and core fluorescence signals was evaluated quantitatively from 100 cells per sample by using the plugin ‘Intensity Correlation Analysis’ of the ‘Image J’ software package (Rasband, W.S., ImageJ, U. S. National Institutes of Health, Bethesda, Maryland, USA; http://imagej.nih.gov/ij/, 1997–2014.). Deconvolution of image z-stacks was performed based on a theoretical point spread function by using the web interface of Huygens Remote Manager (v. 3.2, Scientific Volume Imaging BV). The 3D projections of deconvolved images were reconstructed using the Imaris 8 image analysis software package (Bitplane, Zurich, Switzerland). For quantification of E2- and core-containing structures per cell, the surface tool of Imaris 8 was applied to each channel with the following filters: manual thresholding of absolute intensity, quality and voxel size. The same values of filters were used to each data set and sample. The number of structures per cell was calculated from 30 3D surface reconstructions per sample.

### RNA *in situ* hybridization and immunofluorescence

Forty-eight hours after HCV transfection, Huh7-hp cells grown in 10 cm-diameter dishes were detached and seeded into 24-well plates containing glass coverslips that had been coated with 0.01% (w/v) of Poly-L-Lysin (Sigma P8920). One day after seeding, cells were washed with PBS and fixed with 4% paraformaldehyde for 30 min at RT and permeabilized for 1 h with 70% ice-cold ethanol at 4°C. Permeabilized cells were extensively washed with PBS and HCV RNA positive-strand was detected by FISH using the QuantiGene ViewRNA ISH Cell assay (Affymetryx) according to the instructions of the manufacturer. Immunostaining was performed by blocking the cells at RT for 30 min with 20% (w/v) of goat serum in PBS containing 0.01% Triton X-100, followed by 1 h incubation at RT with the C-830 antiserum (dilution 1:200). Cells were washed three times with PBS and incubated for 1 h in the dark with a 1:1,000 dilution of Alexa 488 secondary antibody (Invitrogen, Molecular Probes). Antibodies were diluted in PBS containing 10% goat serum. Cells were washed three times with PBS and mounted on glass slides with Vectashield (Vector Laboratories Inc.). The JACoP plugin [77] of Image J was used to determine the Mander’s overlap coefficients M_1_ and M_2_, after applying equal thresholds to each channel of 20 images per HCV sample. M_1_ calculates the fraction of red pixels (+ strand HCV RNA) overlapping with green pixels (core protein) and M_2_ the fraction of green pixels (core protein) overlapping with red pixels (+ strand HCV RNA). Deconvolution of images was performed as described above.

### Statistical analysis

Statistical analysis was performed with the GraphPad Prism 5 software package (GraphPad Software, Inc., La Jolla, CA). Significance values were calculated by applying the two-tailed, unpaired Student’s *t* test. *P* values of less than 0.05 were considered statistically significant and the following denotations were used: ***, *P ≤ 0*.001; **, *P ≤ 0*.01; *, *P ≤ 0*.*05; not significant (NS)*, *P ≥0*.*05*.

## Supporting Information

S1 Fig Impact of alanine or glutamic acid substitutions in the basic cluster motif of NS5A DIII on virus production. Quadruple alanine and glutamic acid substitutions specified in the bottom were introduced into the full-length Jc1 genome and transfected into Huh7-Lunet cells. (A) Supernatants were harvested 24, 48 and 72 h after transfection and virus amounts contained in culture supernatants were quantified by limiting dilution assay. Values were normalized to the wildtype (WT) virus that was set to 100%. (B) Four and 48 h post transfection, cells and supernatants were harvested and core amounts were determined by CMIA. For each HCV construct, intra- and extra-cellular amounts of core (normalized to total core amount that was set to 100%) are given (left and right panel, respectively). Mean and SEM of three independent experiments are shown.
(TIF)

S2 Fig Impact of a quadruple mutation affecting the basic cluster motif in NS5A DIII of the genotype 1a isolate H77S on virus production. Glutamic acid residue substitutions (specified in Fig 1A) were introduced into the full-length H77S genome and transfected into Huh7-Lunet cells. Supernatants were harvested 48 and 72 h after transfection and virus amounts contained in culture supernatants were quantified by limiting dilution assay. Values obtained by the H77S basic cluster mutant (BCM) were normalized to the wildtype (WT) virus that was set to 100%. Mean and SEM of two independent experiments are shown.
(TIF)

S3 Fig Properties of virus particles produced by the NS5A basic cluster mutant. (A) A schematic of the Jc1-derived genome containing a Flag-tag at the N-terminus of E2 is given on the top. Huh7-Lunet cells were transfected with the E2-Flag-tag HCV wildtype (WT) genome or the analogous basic cluster mutant (BCM) and 72 h later concentrated supernatants were used for Flag-specific immunoprecipitation. Input (I) and captured (C) Flag peptide-eluted particles were used to quantify HCV RNA amounts by RT-qPCR (left), core protein amounts by core-specific CMIA (middle) and titers of infectious virus by limiting dilution assay (TCID_50_/ml; right). (B, C) Specific infectivities of captured particles were determined by calculating the ratio of TCID_50_ per pg core protein (B) and TCID_50_ per HCV RNA copies (C). Mean and SEM of three independent experiments are shown.
(TIF)

S4 Fig Establishment of experimental conditions to disrupt NS5A-Core interaction. Huh7-Lunet cells were transfected with a Jc1 genome containing a HA tag inserted into NS5A Domain II (see Fig 1A). Seventy two hours later cells were lysed by using two buffers (B1 and B2) with different stringency (see materials and methods). NS5A was enriched by immunoprecipitation (IP) using a HA-specific monoclonal antibody covalently linked to agarose beads. Captured proteins were separated by electrophoresis into an 8% (NS5A) or 15% (Core) acrylamide gel and analyzed by Western blot using antibodies with specificities indicated in the right. Given amounts of input proteins were loaded onto the gel in parallel. Note that B1 maintains NS5A-Core interaction while B2 disrupts this interaction and therefore was used to determine NS5A- and Core-RNA coprecipitation.
(TIF)

S5 Fig Kinetics of replication and particle production of the NS5A FLAG-tagged full length Jc1 genome. (A) Replication kinetics of a full-length Jc1 genome containing a Flag tag inserted at amino acid position 261 of NS5A Domain II (cf. Fig 8). Huh7-Lunet cells were lysed at 4, 24, 48 and 72 h post transfection and core amounts were determined by CMIA. Core amounts were normalized to their respective 4 h-value reflecting transfection efficiency. (B) Quantification of total core protein amounts contained in transfected cells or released into culture supernatants (intra and extracellular: upper and bottom panels, respectively). (C) Kinetic of virus release from Huh7-Lunet cells transfected with full-length genomes specified on the top. Titers of infectious virus were determined by limiting dilution assay. Mean and SEM of three independent experiments are shown.
(TIF)
