# Supplementary figures and images for: Coordination of Hepatitis C Virus Assembly by Distinct Regulatory Regions in Nonstructural Protein 5A
Source: PLoS Pathog. 2016 Jan 4;12(1):e1005376. doi: 10.1371/journal.ppat.1005376 (PMC4699712; doi:10.1371/journal.ppat.1005376)

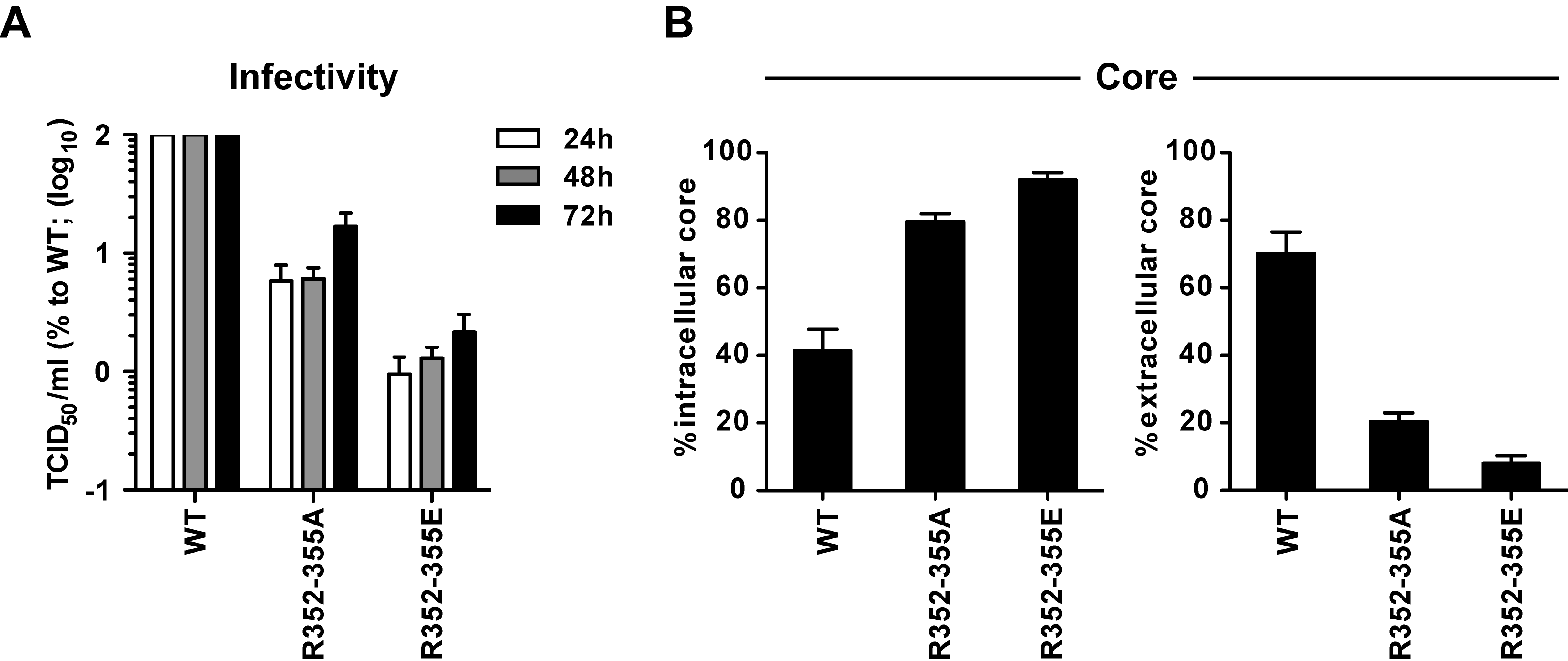

Supplement: S1 Fig — Quadruple alanine and glutamic acid substitutions specified in the bottom were introduced into the full-length Jc1 genome and transfected into Huh7-Lunet cells. (A) Supernatants were harvested 24, 48 and 72 h after transfection and virus amounts contained in culture supernatants were quantified by limiting dilution assay. Values were normalized to the wildtype (WT) virus that was set to 100%. (B) Four and 48 h post transfection, cells and supernatants were harvested and core amounts were determined by CMIA. For each HCV construct, intra- and extra-cellular amounts of core (normalized to total core amount that was set to 100%) are given (left and right panel, respectively). Mean and SEM of three independent experiments are shown. (TIF) [file ppat.1005376.s001.tif]

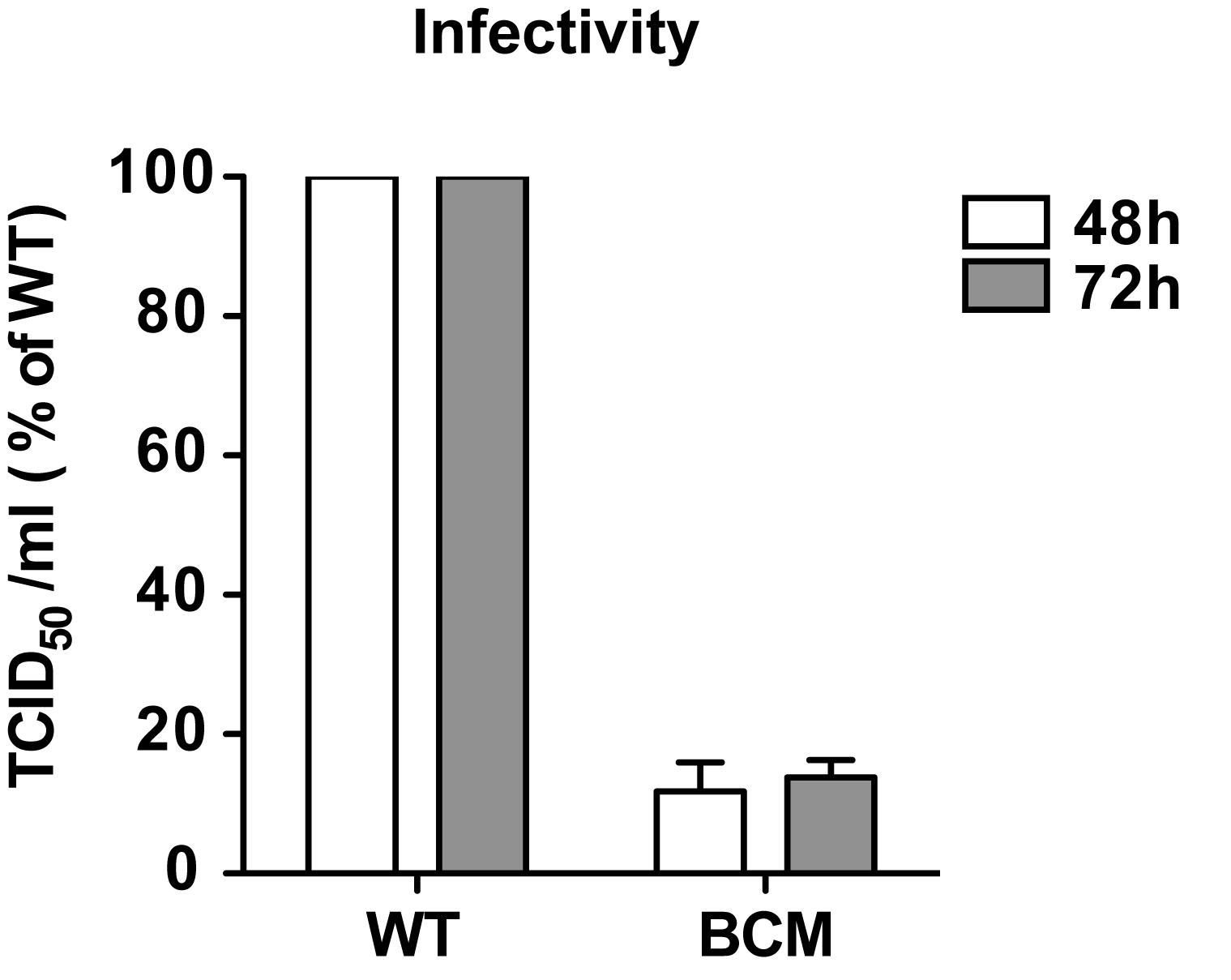

Supplement: S2 Fig — Glutamic acid residue substitutions (specified in Fig 1A) were introduced into the full-length H77S genome and transfected into Huh7-Lunet cells. Supernatants were harvested 48 and 72 h after transfection and virus amounts contained in culture supernatants were quantified by limiting dilution assay. Values obtained by the H77S basic cluster mutant (BCM) were normalized to the wildtype (WT) virus that was set to 100%. Mean and SEM of two independent experiments are shown. (TIF) [file ppat.1005376.s002.tif]

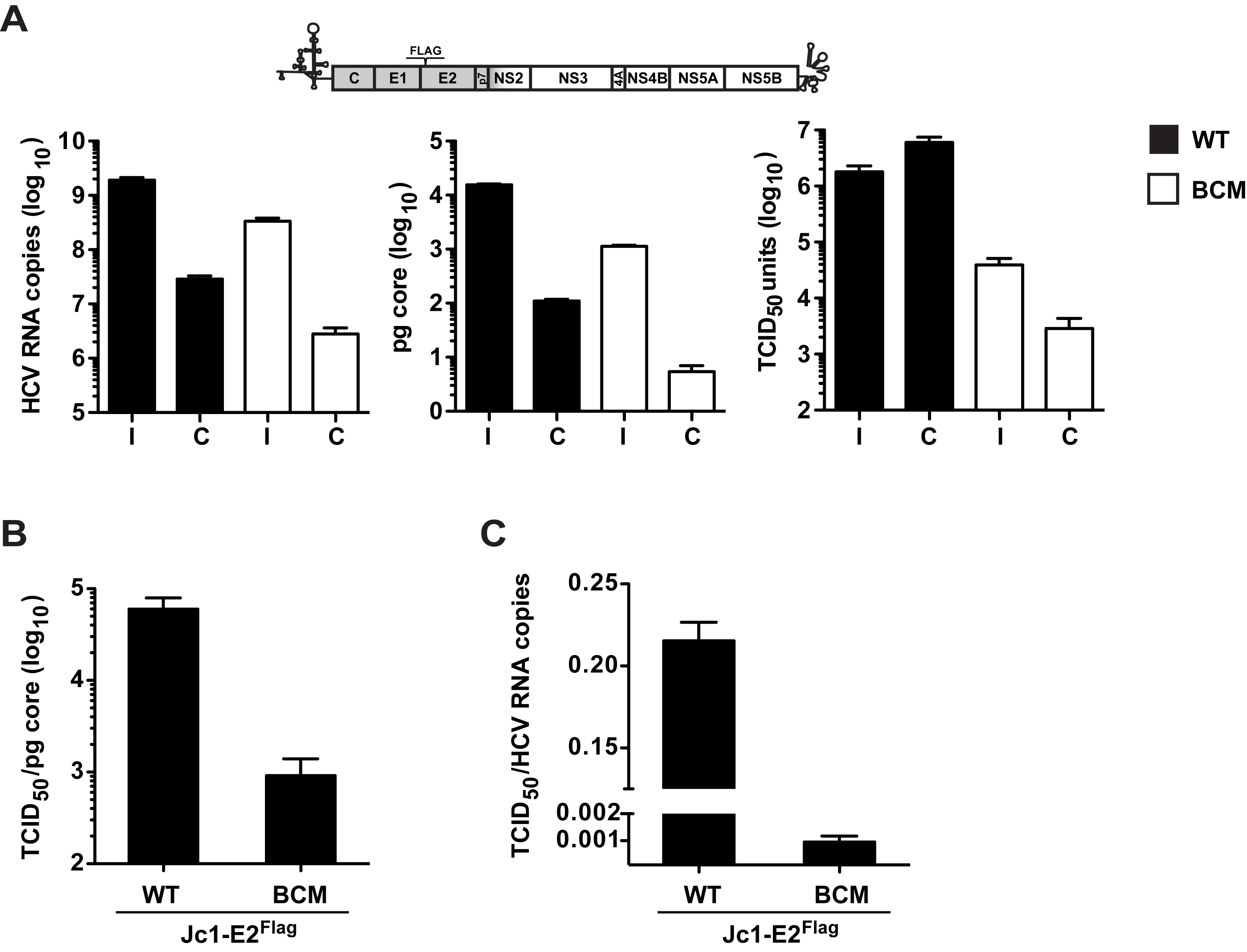

Supplement: S3 Fig — (A) A schematic of the Jc1-derived genome containing a Flag-tag at the N-terminus of E2 is given on the top. Huh7-Lunet cells were transfected with the E2-Flag-tag HCV wildtype (WT) genome or the analogous basic cluster mutant (BCM) and 72 h later concentrated supernatants were used for Flag-specific immunoprecipitation. Input (I) and captured (C) Flag peptide-eluted particles were used to quantify HCV RNA amounts by RT-qPCR (left), core protein amounts by core-specific CMIA (middle) and titers of infectious virus by limiting dilution assay (TCID50/ml; right). (B, C) Specific infectivities of captured particles were determined by calculating the ratio of TCID50 per pg core protein (B) and TCID50 per HCV RNA copies (C). Mean and SEM of three independent experiments are shown. (TIF) [file ppat.1005376.s003.tif]

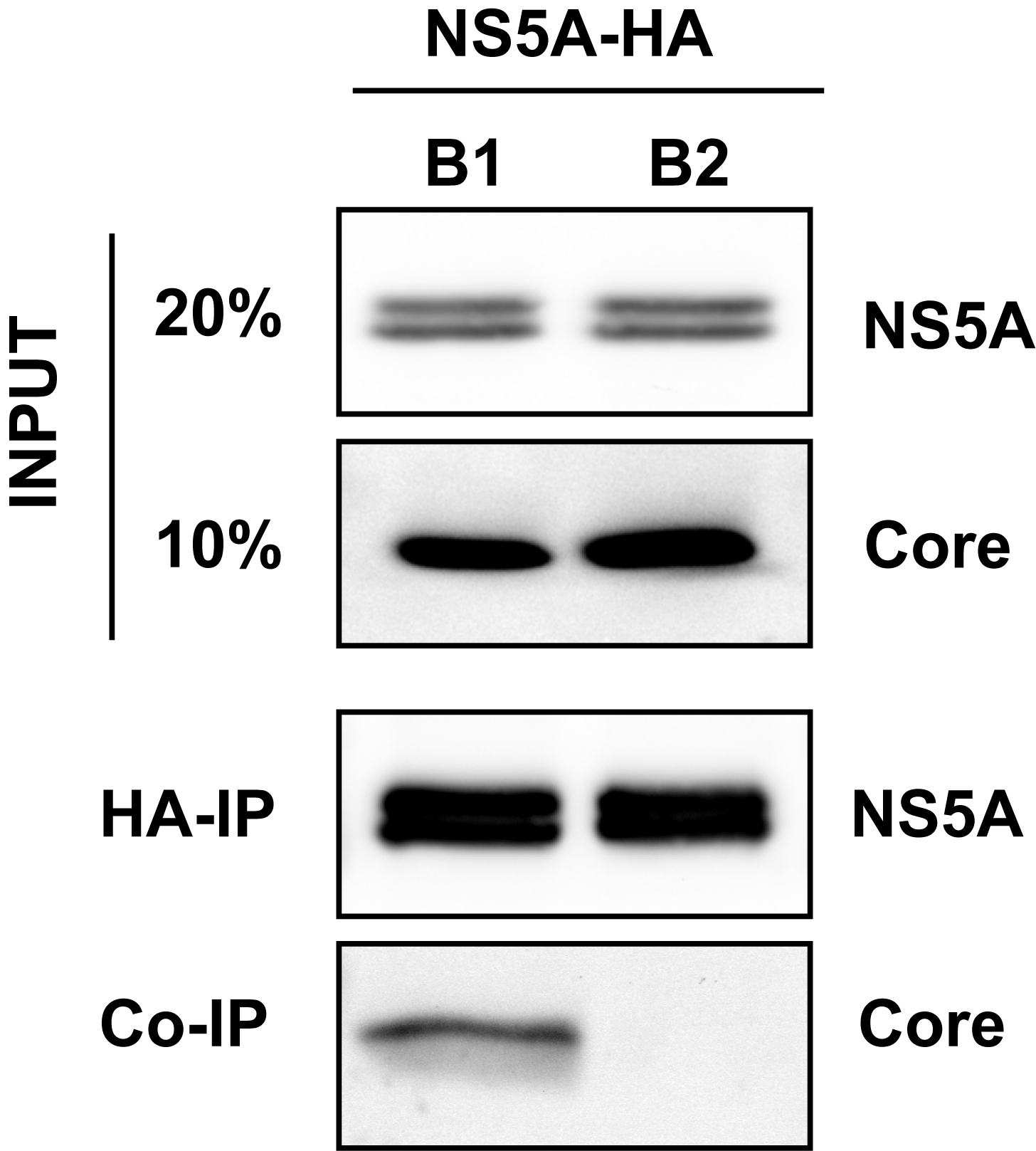

Supplement: S4 Fig — Huh7-Lunet cells were transfected with a Jc1 genome containing a HA tag inserted into NS5A Domain II (see Fig 1A). Seventy two hours later cells were lysed by using two buffers (B1 and B2) with different stringency (see materials and methods). NS5A was enriched by immunoprecipitation (IP) using a HA-specific monoclonal antibody covalently linked to agarose beads. Captured proteins were separated by electrophoresis into an 8% (NS5A) or 15% (Core) acrylamide gel and analyzed by Western blot using antibodies with specificities indicated in the right. Given amounts of input proteins were loaded onto the gel in parallel. Note that B1 maintains NS5A-Core interaction while B2 disrupts this interaction and therefore was used to determine NS5A- and Core-RNA coprecipitation. (TIF) [file ppat.1005376.s004.tif]

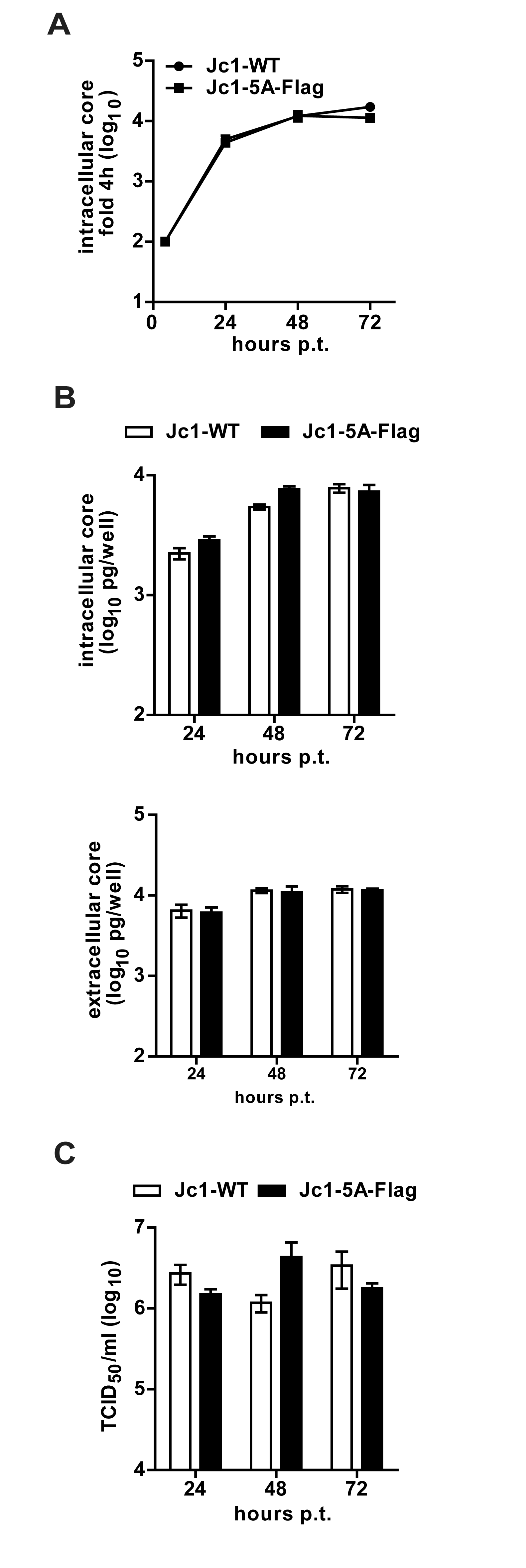

Supplement: S5 Fig — (A) Replication kinetics of a full-length Jc1 genome containing a Flag tag inserted at amino acid position 261 of NS5A Domain II (cf. Fig 8). Huh7-Lunet cells were lysed at 4, 24, 48 and 72 h post transfection and core amounts were determined by CMIA. Core amounts were normalized to their respective 4 h-value reflecting transfection efficiency. (B) Quantification of total core protein amounts contained in transfected cells or released into culture supernatants (intra and extracellular: upper and bottom panels, respectively). (C) Kinetic of virus release from Huh7-Lunet cells transfected with full-length genomes specified on the top. Titers of infectious virus were determined by limiting dilution assay. Mean and SEM of three independent experiments are shown. (TIF) [file ppat.1005376.s005.tif]
